# Subregional differences in the hippocampal transcriptomic response after penetrating traumatic brain injury in rats

**DOI:** 10.3389/fneur.2025.1729794

**Published:** 2026-02-23

**Authors:** Erik Lidin, Mårten Risling, Mattias K. Sköld

**Affiliations:** 1 Experimental Traumatology Unit, Department of Neuroscience, Karolinska Institutet, Stockholm, Sweden; 2 Section of Neurosurgery, Department of Medical Sciences, Uppsala University Hospital, Uppsala, Sweden

**Keywords:** cornu ammonis, dentate gyrus, hippocampus, neuroinflammation, penetrating traumatic brain injury, RNA-seq, traumatic brain injury

## Abstract

Penetrating traumatic brain injuries, often caused by projectiles like shrapnel, have become increasingly common in modern warfare. These injuries have high mortality rates and can lead to severe, long-term neurological deficits. The hippocampus is composed of distinct subregions with unique transcriptomic profiles and cytoarchitecture, and its dysfunction after TBI is closely linked to neurological sequelae, including cognitive and memory impairments. While previous research has explored general brain responses to TBI, the specific molecular changes in individual hippocampal subregions in TBI remain poorly understood. To address this, we used laser-capture microdissection, RNA-sequencing, and differential gene expression matched with gene ontology analysis to investigate transcriptional responses in hippocampal subregions (CA1, CA2, CA3, and dentate gyrus) following high-velocity penetrating TBI in a rat model. Our findings reveal distinct gene expression patterns in each region, reflecting varied pathophysiological responses. CA1 exhibited increased expression of cell-cycle and gliogenesis-associated genes, indicating cytoskeletal stress and gliogenesis-associated signaling. CA2 showed strong immune activation, highlighting leukocyte signaling, MHC antigen processing, and complement pathways, coupled with downregulation of oxidative phosphorylation, suggesting immune-driven metabolic dysfunction. CA3 displayed a pronounced inflammatory profile, marked by TNF signaling and adhesion remodeling. In contrast, the dentate gyrus upregulated genes linked to tissue repair, including ECM stabilization and angiogenesis, suggesting a neuroprotective response. These results highlight the complex, subregion-specific balance between injury and repair mechanisms following TBI, with the hippocampus likely contributing to injury progression through its widespread neuronal connections. Understanding these molecular dynamics is essential for developing targeted interventions aimed at mitigating damage and promoting recovery, especially in the context of increasing high-velocity brain injuries due to modern conflict.

## 1 Introduction

Penetrating traumatic brain injury (pTBI) is associated with high mortality and poor long-term outcomes (1). In recent wars, the incidence of penetrating head injuries has significantly increased, mainly from high-velocity projectiles like shrapnel (2). The biomechanical effects of penetrating TBI vary with object velocity and energy. High-velocity injuries induce transient cavitation, shock-wave propagation, and shearing forces, which, together with subsequent secondary injury cascades, expand the tissue damage well beyond the initial wound tract (3). The secondary injury is initiated immediately after the primary insult and involves multifaceted pathological mechanisms, including excitotoxicity, mitochondrial dysfunction, oxidative stress, ischemia, and extensive neuroinflammation (4–8). The secondary injury can persist for months, leading to progressive neuronal death and sequelae development (8–12). Mitigation of secondary injury is therefore a central therapeutic goal for improving injury outcome and limiting sequelae development. Despite this, no pharmacological neuroprotective agent exists to date.

The hippocampus, vital for memory, cognition, and emotional regulation, is particularly vulnerable after TBI (13–15). Post-traumatic sequelae such as cognitive impairment, amnesia, post-traumatic epilepsy, and affective disturbances are closely linked to hippocampal dysfunction (15, 16). The hippocampus is also a particularly suitable model for experimental injury, as its well-established behavioral readouts, such as spatial navigation, contextual memory, and pattern-separation tasks, allow direct assessment of functional consequences and provide platforms for evaluating therapeutic interventions following injury. Structurally, the hippocampus is heterogeneous, comprising distinct subfields CA1, CA2, CA3, and the dentate gyrus (DG). Each subregion is defined by unique cytoarchitecture, connectivity, transcriptional programs, and functional roles, which collectively are believed to influence their selective vulnerabilities to injury (17–20). CA1, the principal hippocampal output, is essential for episodic and spatial memory and is highly vulnerable to ischemia and excitotoxicity, with injury producing marked cognitive deficits (20). CA2, though relatively resistant, is specialized for social memory and shows enrichment in signaling and metabolism-related pathways, features that may influence both its resilience to injury and its contribution to social and affective disturbances after TBI (21–23). CA3, with an excitatory architecture, supports pattern completion and associative recall but is predisposed to hyperexcitability and seizure generation, consistent with its transcriptional enrichment in synaptic and calcium signaling pathways (24). The DG, the hippocampal input gate, mediates pattern separation and adult neurogenesis, supported by strong enrichment in ribosomal and biosynthetic programs (23, 25). Following TBI, it is particularly susceptible to gliosis and impaired neurogenesis (26).

While hippocampal subfield transcriptomes are well characterized under physiological conditions, their responses to injury are far less understood (23, 27, 28). Using in-situ hybridization, we have previously shown that hippocampal subregions display distinct gene regulatory responses to pTBI, with marked region-dependent differences (29). Next-generation sequencing studies have further provided insight into hippocampal pathophysiology after TBI, revealing transcriptomic changes linked to secondary injury in whole hippocampi or selected subfields (30–32). However, such approaches lack spatial and anatomical resolution, either averaging across subregions or omitting neighboring areas, thereby risking the loss of diverse or coordinated subregional responses.

Although global hippocampal transcriptomic alterations after TBI have been reported, subregion-specific responses remain underexplored. Our previous work showed that hippocampal subfields regulate genes independently after pTBI, highlighting the need for high-throughput, subfield-resolved analyses (29). To our knowledge, this has not been systematically addressed. Yet such resolution is essential for disentangling mechanisms of secondary injury, including neuroinflammation, gliosis, metabolic collapse, impaired regeneration, and for identifying therapeutic targets. To address this gap, we applied laser-capture microdissection (LCMD) and RNA sequencing to isolate and profile hippocampal subregions in a rat model of high-velocity pTBI during the subacute injury phase. This approach provides high-resolution insight into subregional molecular heterogeneity and reveals signaling pathways that exacerbate or mitigate injury progression within a probable therapeutic window. By comparing subregions, we aim to define the distinct transcriptional signatures associated with immune activation, metabolic disruption, and neuroprotective remodeling in the hippocampus after penetrating TBI to enable targeted treatment in the future.

## 2 Materials and methods

All procedures were approved by the Swedish Regional Ethics Approval Board for animal research under license 7965-2022.

A total of 8 adult male Sprague–Dawley rats were assigned to sham surgery (*n* = 4) or penetrating traumatic brain injury (pTBI) (*n* = 4), with a survival time of 72 h. The mean weight for sham was 334 g (SD = 2.9 g), and for pTBI 339 g (SD = 14.9 g). Animals were housed pairwise in standard-sized cages, under a 12-h light/dark cycle with ad libitum access to water and pellets.

### 2.1 Surgery and penetrating trauma

All animals were anesthetized in a sealed chamber containing 4% isoflurane and subsequently placed on an autoregulated heat pad within a stereotactic frame. They were then maintained under 2% isoflurane via a stereotactic nose mask (Kopf Instruments, no: 906). Analgesia was provided with buprenorphine (0.03 mg/kg) and 1.5 mL of Ringer’s acetate, administered subcutaneously, along with a 50/50 mixture of Lidocaine and Marcaine (0.250 mg/mL) applied to the scalp. A midline incision was made through the skin and periosteum, followed by a 2.75 mm burr hole drilled 2 mm posterior and 2 mm lateral to bregma, exposing the dura. The secondary projectile was positioned adjacent to the dura. The secondary projectile consisted of a 30 mm long aluminum probe with a spherical tip (diameter 2 mm), weighing 0.66 g. A modified air rifle (CNC-Process AB, Hova, Sweden) was used to fire a lead pellet, which impacted the secondary projectile that accelerated to a speed of 110 m/s, penetrating the meninges and brain parenchyma. The spherical tip causes a direct tissue laceration but also produces a surrounding injury zone due to transient cavity expansion, propagating a pressure wave into adjacent tissue and resulting in damage that extends beyond the immediate laceration site (33). A cone-shaped section of the secondary probe served as a stopper, limiting the mean penetration depth to 5.075 mm (*n* = 4, SD = 0.05 mm) and ensuring survivability. In the sham surgery group, a blank bullet was used, thus no penetration occurred.

Lastly, the skin was sutured, and animals were placed in a recovery box on an autoregulated heat pad and supplied 100% O_2_ until mobile. Buprenorphine (0.03 mg/kg) was administered for analgesia to all animals, regardless of intervention, three times daily for three days post-surgery. Previous studies describing the penetration model in further depth are available (33–35).

### 2.2 Tissue collection

Rats were sacrificed by guillotine decapitation under isoflurane anesthesia 72 h after pTBI or sham induction. Brains were harvested and flash-frozen on dry ice, then embedded in OCT and stored at −80 °C. No tissue perfusion was performed, in order to minimize the interval between animal sacrifice and tissue freezing to preserve RNA quality. 14 μm sections were acquired at Bregma: – 3.60 mm (Paxinos 6th edition) using a CryoStar NX70 cryostat treated with RNaseZap (Invitrogen, Cat no: AM9780) to eliminate RNase contamination. Sections intended for LCMD and RNA-sequencing were placed on MMI membrane slides (MMI, Cat no: 50102), and directly adjacent sections used for multiplex immunofluorescence on SuperFrost Plus slides (Thermo Fisher Scientific).

### 2.3 Modified cresyl violet staining

All ethanol solutions were diluted from 100% using RNase-free water (Invitrogen, cat no: 10977035) in a RNase-free environment. Sections intended for LCMD and subsequent RNA sequencing were fixed for 5 min in −20 °C 70% ethanol and then rinsed twice for 10 s in RNase-free PBS (Invitrogen, cat no: AM9624) before nuclear staining with a modified cresyl violet solution (1% w/w in 75% ethanol) for 20 s. Afterward, sections were dehydrated in 70% ethanol and 100% ethanol for 30 s and 1 min, respectively. Following dehydration, sections were incubated at room temperature for 15 min. Sections were prepared one at a time and dissected immediately after the 15-min incubation period.

### 2.4 Laser-capture microdissection (LCMD)

**Figure 1:**
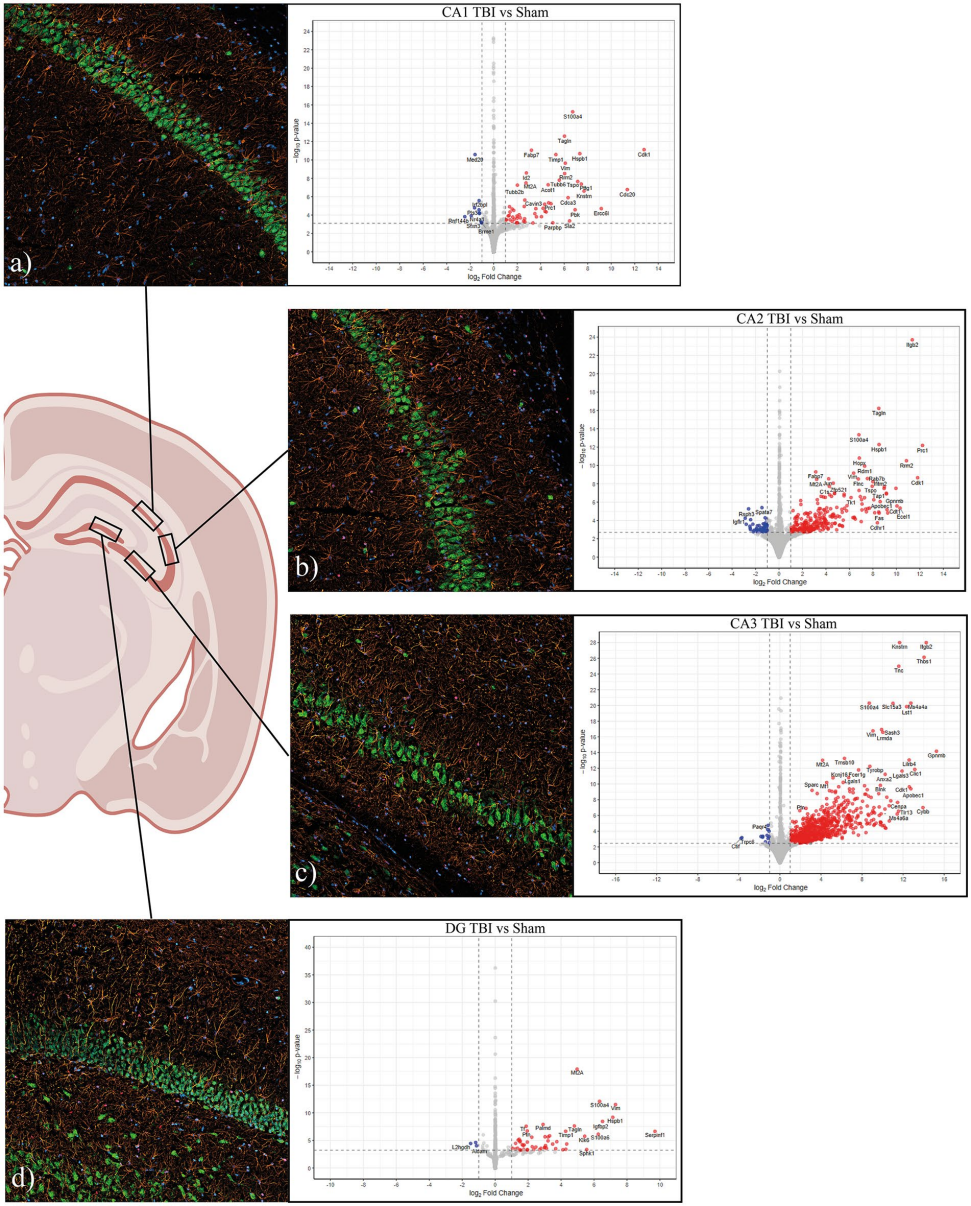
Schematic overview of hippocampal subregion sampling and transcriptional responses following TBI. Schematic illustration showing the hippocampal section (Bregma −3.6 mm) and the standardized subregional sampling locations [CA1 **(a)**, CA2 **(b)**, CA3 **(c)**, DG **(d)**] used for laser-capture microdissection (LCMD). Representative immunofluorescence images of the captured regions **(a–d)** show local cellular composition with DAPI (nuclei, blue), NeuN (neurons, green), GFAP (astrocytes, orange), and Olig2 (oligodendrocytes, red) labeling, confirming clear neuronal layer delineation and low glial contamination, primarily consisting of astrocytic processes. Adjacent panels display corresponding volcano plots illustrating differential gene expression (DGE) between TBI and sham for each subregion. Analyses were performed using DESeq2 v1.44.0 with log_2_ fold-change shrinkage (apeglm) and Benjamini–Hochberg multiple-testing correction. Genes with adjusted *p* < 0.05 and log_2_ fold-change > 1 were considered significant. The *x*-axis represents log₂ fold change, and the *y*-axis shows –log₁₀ adjusted *p*-values, with red and blue points indicating significantly upregulated and downregulated genes, respectively. Across subregions, a predominance of upregulated genes was observed, with the most extensive transcriptional activation in CA2 and CA3, reflecting pronounced subregion-specific injury responses. LCMD was consistently performed at the same anatomical coordinates in each animal to ensure regional comparability. The image was partially created in BioRender. CA, cornu ammonis; DG, dentate gyrus; DGE, differential gene expression; LCMD, laser-capture microdissection; TBI, traumatic brain injury.

**Figure 2:**
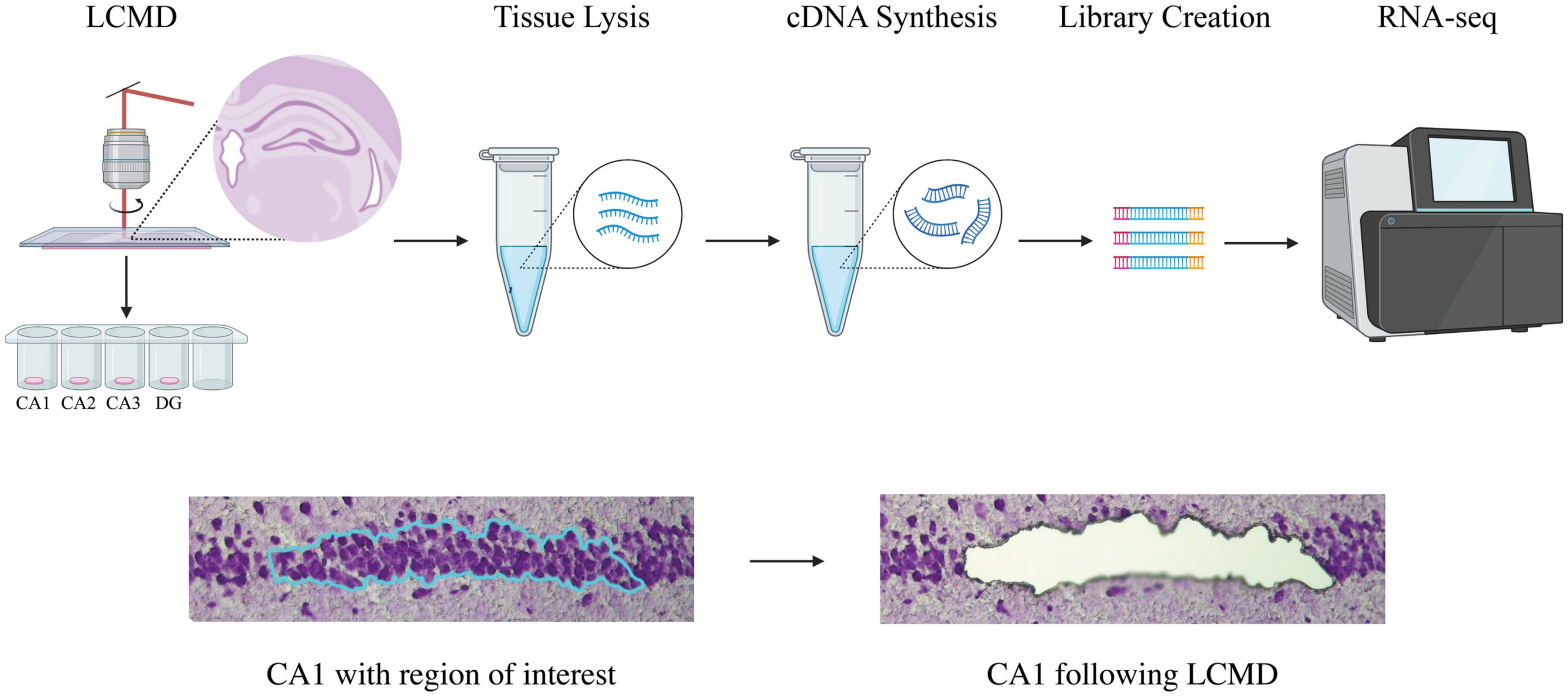
Workflow overview from laser-capture microdissection to RNA sequencing. Schematic overview of the experimental workflow from laser-capture microdissection (LCMD) to RNA sequencing (RNA-seq). LCMD was used to isolate the pyramidal or granule cell layer from hippocampal subregions (CA1, CA2, CA3, DG), followed by tissue lysis, cDNA synthesis, library preparation, and sequencing. The lower panels illustrate a representative CA1 section stained with cresyl violet before and after LCMD, showing the precisely defined region of interest (blue) corresponding to the pyramidal layer and the high spatial specificity of the laser dissection, with the targeted tissue used for downstream analysis fully removed post-capture. The image was partially created in BioRender. CA, cornu ammonis; DG, dentate gyrus; LCMD, laser-capture microdissection; RNA-seq, RNA sequencing.

LCMD was conducted using a Leica DM6000B microscope equipped with a Leica LMD7000 laser-capture system. Hippocampal subregions were dissected at predefined locations described in Figure 1. Dissections were performed at 20x magnification, carefully delineating the pyramidal layer for CA1-CA3 and the granular layer for the DG to minimize glial contamination. Approximately 100 neurons were collected from each sample and subregion. The dissected tissue was collected into 0.2 mL adhesive caps (Zeiss, Cat no: 415190–9,181-000), and 8 μL of nuclease-free water, along with 1 μL of 10x Reaction Buffer (Takara Bio, Cat no: 635013), was added for initial lysis. Following a 1-min centrifugation, the samples were placed on dry ice and subsequently stored at −80 °C. In order to minimize potential batch effects, tissue sections were thawed, stained, and microdissected individually one at a time using a standardized workflow to ensure comparable handling times from thawing to lysis and freezing, with samples processed in randomized order. Figure 2 presents a schematic overview of the sample processing pipeline and images illustrating the specificity of the LCMD procedure.

### 2.5 RNA-sequencing

SMART-Seq libraries were synthesized from total RNA using SMART-Seq v4 Ultra Low Input RNA Kit (Takara Bio) according to the manufacturer’s instructions. The first-strand cDNAs were synthesized from the total RNA using nontemplated nucleotides added by the SMARTScribe Reverse Transcriptase, then, the double-strand cDNAs were synthesized by template switching. PCR was directly performed and samples purified. Quality control of cDNAs was performed on Agilent Tapestation according to the manufacturer’s instructions. 1 ng of cDNA was used for the tagmentation reaction. During tagmentation, samples are fragmented and tagged with adapters, followed by PCR amplification and index addition. The indexed libraries were purified, normalized, combined, and sequenced on the Illumina NovaSeq X Plus 10B-300 lane generating 2×150 bp paired-end reads. Basecalling and demultiplexing was performed using bcl2fastq software with default settings generating Fastq files for further downstream mapping and analysis. Library preparation was performed in a blinded and randomized manner, and all libraries were sequenced together in a single run, minimizing batch-related effects on subfield-level clustering.

### 2.6 Immunofluorescence

Sections used for immunofluorescent staining were directly adjacent to the sections used for LCMD and subsequent RNA-sequencing. Sections were thawed for 20 min at room temperature, fixed in 4% paraformaldehyde for 10 min at 4 °C, and washed in PBS for 15 min. Slides were incubated with blocking solution (0.01 M PBS + 0.1% NaN3 + 0.3% Triton + 5% BSA) for 45 min at room temperature, followed by incubation with primary antibodies toward microglia (rabbit anti-Iba1, FUJIFILM, cat no: 019–19,741, 1:500), neurons (chicken anti-NeuN, Sigma, cat no: ABN91, 1:1000), astrocytes (rabbit anti-GFAP, Abcam, cat no: ab33922, 1:1000), oligodendrocytes (goat anti-OLIG2, R&D Systems, AF2418, 1:500), leukocytes (mouse anti-CD45, Abcam, cat no: ab33923, 1:50), oligodendrocyte precursors (mouse anti-NG2, Millipore, cat no: MAB5384-I, 1:50), epithelial tight junctions/vessels (rabbit anti-Occludin, Abcam, cat no: ab216327, 1:200), in blocking solution for 16 h at 4 °C. The next day, slides were washed in PBS for 30 min and incubated with secondary antibodies (Alexa Fluor, all cross-adsorbed, donkey anti-mouse 555 & 647, anti-goat 488, anti-rabbit 555 & 647, all 1:500) in a buffer (0.01 M PBS + 0.1% NaN3 + 0.3% Triton) for 45 min at room temperature. Slides were washed 30 min in PBS, incubated with DAPI for 10 min, washed again in PBS for 30 min, and mounted with Prolong Diamond antifade mounting medium (Invitrogen).

### 2.7 Image acquisition and processing

Images for immunofluorescent analysis were acquired using a Zeiss LSM800 confocal microscope at 20x magnification, with four images captured per subregion and subsequently arranged as 2 × 2 tiles. Images were processed in Fiji/ImageJ, with automated contrast adjustment followed by channel merging and background subtraction as no intensity quantification was performed. Images were analyzed qualitatively, assessing infiltration and presence of non-neuronal integration of the pyramidal and granule cell layer.

### 2.8 Statistics and bioinformatics

RNA-seq data was processed using a standard analysis pipeline. Basecalling and demultiplexing was performed with bcl2fastq (v2.20.04.422). Reads were aligned to the Ensembl *Rattus norvegicus* reference genome (mRatBN7.2) using STAR (v2.7.9a). Gene-level counts were generated with featureCounts (v1.5.1) using Ensembl annotations. Downstream normalization, annotation, visualization and differential gene expression (DGE) analysis were conducted using DESeq2 (v1.44.0). For DGE, significance was assessed using a Wald test between experimental groups. Log_2_ fold-change shrinkage was applied with apeglm, and *p*-values were adjusted for multiple comparisons using the Benjamini–Hochberg method. Genes were considered significantly differentially expressed when adjusted *p* < 0.05 and the log_2_ fold-change > 1.

Gene set enrichment analysis (GSEA) was conducted using fgsea with gene sets derived from the Gene Ontology (GO) knowledgebase. Parameters included a minimum gene set size of 15 and a maximum of 500, with significance set at *p* < 0.005 after Benjamini–Hochberg correction. Over-representation analysis (ORA) was likewise performed using GO terms, using ClusterProfiler. Input genes were limited to those meeting the significance criteria (adjusted *p* < 0.05, log_2_ fold-change > 1), up to 1,000 genes. Gene sets containing between 15 and 500 members were tested, and enrichment *p*-values were adjusted via the Benjamini–Hochberg method.

## 3 Results

### 3.1 Immunofluorescence

**Figure 3:**
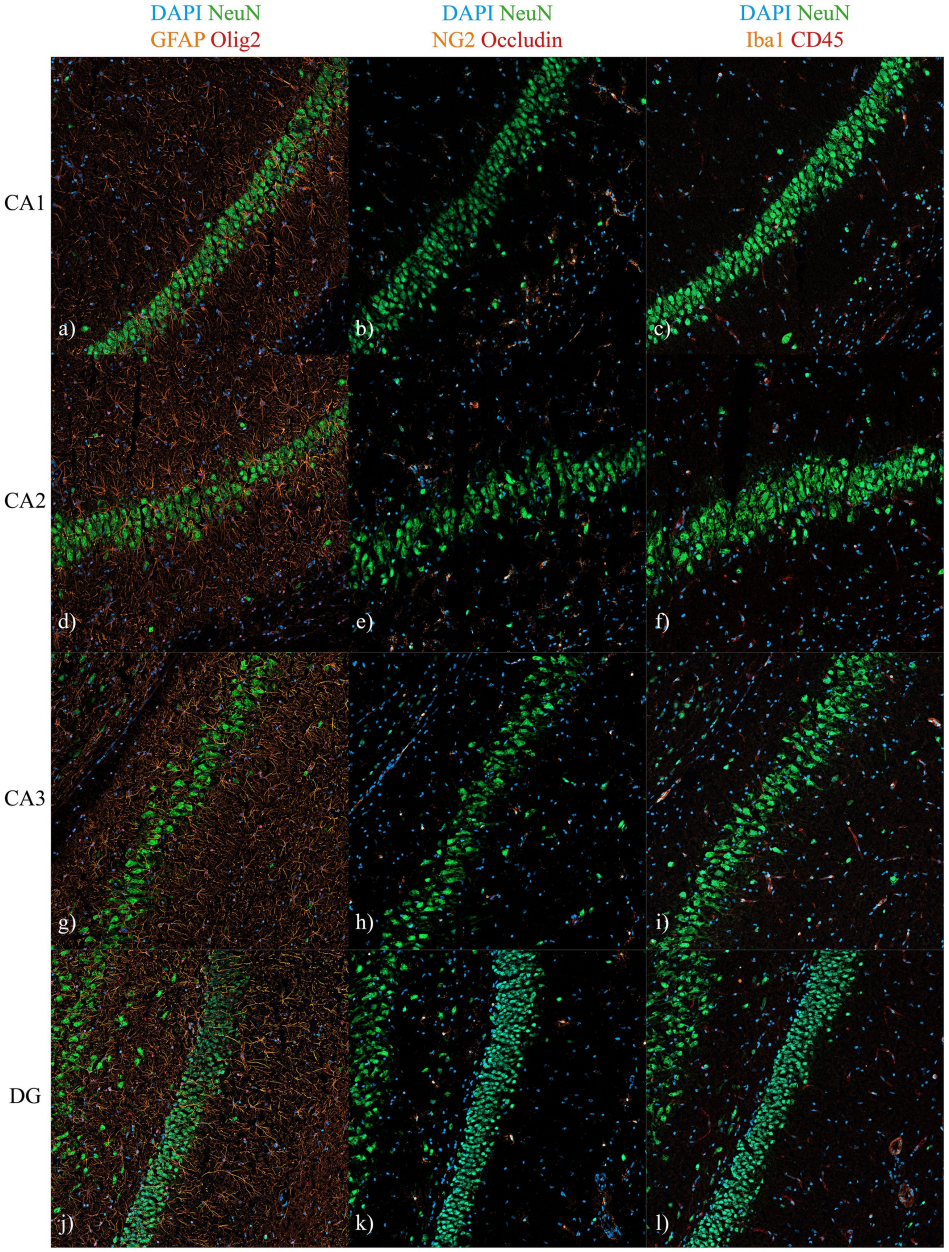
Cellular composition of hippocampal subregions surrounding the pyramidal and granule cell layers 72 h post-injury. Representative immunofluorescence images (20 × magnification, 2 × 2 tile scans) from hippocampal subfields (CA1–CA3, DG) located at Bregma −3.6 mm from animals subjected to penetrating TBI. Sections were stained for DAPI (nuclei), NeuN (neurons), GFAP (astrocytes), Olig2 (oligodendrocytes), NG2 (oligodendrocyte progenitors), Occludin (endothelial tight junctions/vessels), Iba1 (microglia), and CD45 (infiltrating immune cells). The aim was to assess local cellular composition around the pyramidal and granule cell layers and evaluate potential non-neuronal contamination in the laser-captured regions. NeuN staining confirmed the strong neuronal specificity of these layers, with only sparse GFAP-positive processes suggesting low astrocytic contribution. No Olig2-, NG2-, Iba1-, or CD45-positive cells were detected within the neuronal layers, indicating negligible contamination by oligodendrocytes, OPCs, microglia, or infiltrating immune cells. Occludin labeling was confined to perivascular regions outside the dissected zones. Images are representative of areas used for laser-capture microdissection and derived from immediately adjacent 14 μm sections. Larger images are available in Supplementary Figures 1–3. CA, cornu ammonis; DG, dentate gyrus; TBI, traumatic brain injury.

The pyramidal layers of CA1–CA3 and the granule layer of the DG were predominantly composed of NeuN-positive neurons. Limited glial staining was detected within the pyramidal layer, mainly corresponding to distal processes of GFAP-positive astrocytes. No hypertrophy of astrocytic processes was observed. Iba1- and CD45-positive cells were absent from the pyramidal layer, Olig2 staining was sparse overall, and occludin expression was confined to regions outside the pyramidal layer. NG2-positive cells were observed only in co-localization with occludin and were therefore not classified as oligodendrocytes; no NG2-positive but occludin-negative cells were detected. A representative panel illustrating the cellular distribution across hippocampal subregions is shown in Figure 3.

### 3.2 Sequencing quality control

The mean number of total reads per sample ranged from 43.4 to 51.9 million, with consistently high mapping and assignment rates. Three samples were excluded during quality control based on objective sequencing and assignment metrics prior to analysis: one DG TBI sample due to insufficient sequencing depth (5.1 million total reads), and two sham samples due to markedly low numbers of assigned reads despite adequate total read counts, consistent with suspected technical or contamination-related issues (CA3 sham: 46.0 million reads, 3.49 million assigned; CA2 sham: 46.8 million total reads, 5.19 assigned). These excluded samples originated from different subregions and experimental groups and therefore did not disproportionately affect any specific region.

**Figure 4:**
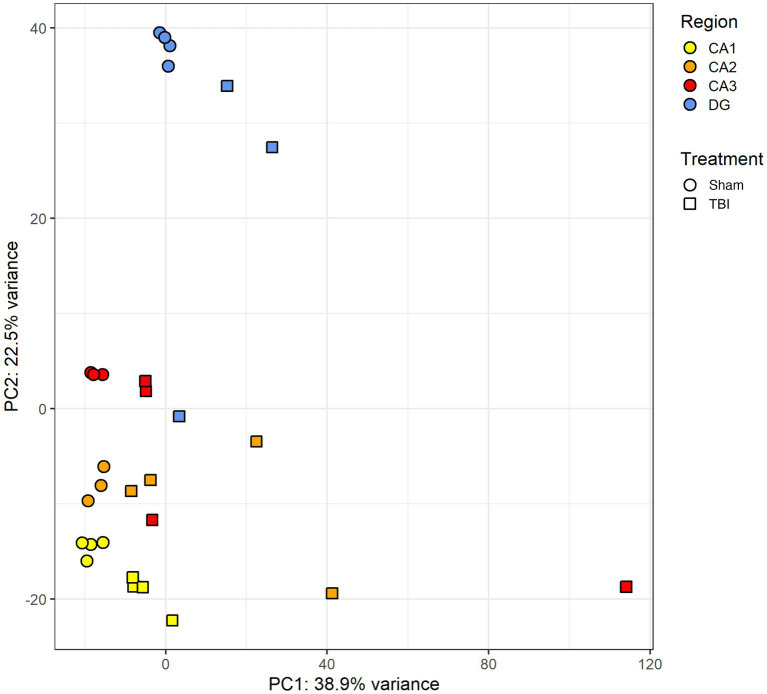
Principal component analysis of hippocampal subregion transcriptomes following penetrating TBI. Principal component analysis (PCA) plot based on the 1,000 most variable genes from variance-stabilized (VSD) RNA-seq data of hippocampal subregions (CA1–CA3, DG) in sham and TBI animals 72 h post-injury. Each point represents an individual sample, colored by subregion and shaped by condition (circle = sham, square = TBI). The first two principal components explain 38.9% (PC1) and 22.5% (PC2) of the total variance. Samples cluster by both subregion and intervention, reflecting spatial and injury-related transcriptional segregation. CA1 (Sham *n* = 4, TBI *n* = 4), CA2 (Sham *n* = 3, TBI *n* = 4), CA3 (Sham *n* = 3, TBI *n* = 4), and dentate gyrus (DG; Sham *n* = 4, TBI *n* = 3). CA, cornu ammonis; DG, dentate gyrus; PCA, principal component analysis; TBI, traumatic brain injury; VSD, variance-stabilized data.

In CA1, sham samples yielded an average of 43.5 (SD = 3.8) million total reads, with 87.1% (SD = 1.8) uniquely mapped and 60.1% (SD = 2.2) assigned to annotated transcripts. TBI samples in CA1 showed comparable sequencing depth, with 43.9 (SD = 6.3) million total reads, 82.9% (SD = 4.2) uniquely mapped and 54.3% (SD = 3.2) assigned. In CA2, total reads averaged 50.0 (SD = 6.4) million for sham and 50.5 (SD = 13.4) million for TBI. In sham, 85.2% (SD = 1.4) were uniquely mapped and 59.6% (SD = 1.4) assigned. For TBI, 83.0% (SD = 1.2) were uniquely mapped and 56.1% (SD = 5.2) assigned. CA3 yielded 45.7 (SD = 1.47) million reads in the sham group and 50.5 (SD = 6.1) million in the TBI group. Mapping and assignment percentages remained consistent across interventions, in sham 86.8% (SD = 1.7) were uniquely mapped and 63.4% (SD = 2.0) assigned, after TBI uniquely mapped and assigned transcripts were 83.3% (SD = 3.0) and 62.1% (SD = 3.8), respectively. In DG, sham had 48.1 (SD = 10.5) million total reads with 85.8% (SD = 2.2) uniquely mapped and 56.9% (SD = 2.7) assigned. TBI samples in DG exhibited similar metrics 49.7 (SD = 1.8) million total reads, 85.5% (SD = 3.2) uniquely mapped, and 53.4% (SD = 5.1) assigned. No substantial differences were observed in sequencing depth or mapping efficiency between sham and TBI groups within any subregion, supporting consistent RNA quality and library preparation across conditions. A summary of dissected tissue size and sequencing quality indicators are presented in Table 1, and principal component analysis (PCA) clustering in Figure 4.

**Table 1:** Group-mean tissue size, sequencing quality metrics, and number of samples included in downstream analyses divided by hippocampal subregion.

Region	Intervention	Size, mm^2^	Reads	Unique, %	Assigned, %	*n* =
CA1	Sham	0.051 (0.006)	43,466,772 (3784542)	87.05 (1.81)	60.14 (2.17)	4
CA1	TBI	0.048 (0.007)	43,912,094 (6297061)	82.85 (4.19)	54.26 (3.21)	4
CA2	Sham	0.046 (0.002)	50,002,974 (6432245)	85.22 (1.40)	59.55 (1.37)	3
CA2	TBI	0.050 (0.005)	50,539,288 (13431064)	83.02 (1.19)	56.12 (5.16)	4
CA3	Sham	0.057 (0.011)	45,650,203 (1474449)	86.78 (1.69)	63.38 (1.96)	3
CA3	TBI	0.047 (0.005)	50,475,964 (6060782)	83.33 (2.98)	62.13 (3.82)	4
DG	Sham	0.051 (0.002)	48,064,214 (10486306)	85.77 (2.23)	56.92 (2.74)	4
DG	TBI	0.052 (0.009)	49,660,549 (1839713)	85.48 (3.16)	53.43 (5.11)	3

All downstream analyses were performed after exclusion of three samples that failed quality control criteria. One dentate gyrus (DG) TBI sample was excluded due to insufficient sequencing depth (5.1 million total reads). Two sham samples were excluded due to low assigned transcripts despite adequate total reads (CA2 sham: 46.8 million total reads with 5.19 million assigned reads; CA3 sham: 46.0 million total reads with 3.49 million assigned reads). SD in parentheses.

### 3.3 Differential gene expression and gene ontology-based analysis

Selection of differentially expressed genes (DEGs) and significance thresholds were set at adjusted *p*-value < 0.05 and a log_2_ fold change (LFC) > 1 or < −1.

#### 3.3.1 CA1

In total, 69 genes were identified as significantly differentially expressed in CA1, with 58 upregulated and 11 downregulated transcripts. The top upregulated genes included Cdk1 (Cyclin-dependent kinase 1, LFC = 12.8), S100a4 (S100 calcium-binding protein A4, LFC = 6.7), Hspb1 (Heat shock protein family B member 1, LFC = 7.32), Tspo (Translocator protein, LFC = 7.15).

**Figure 5:**
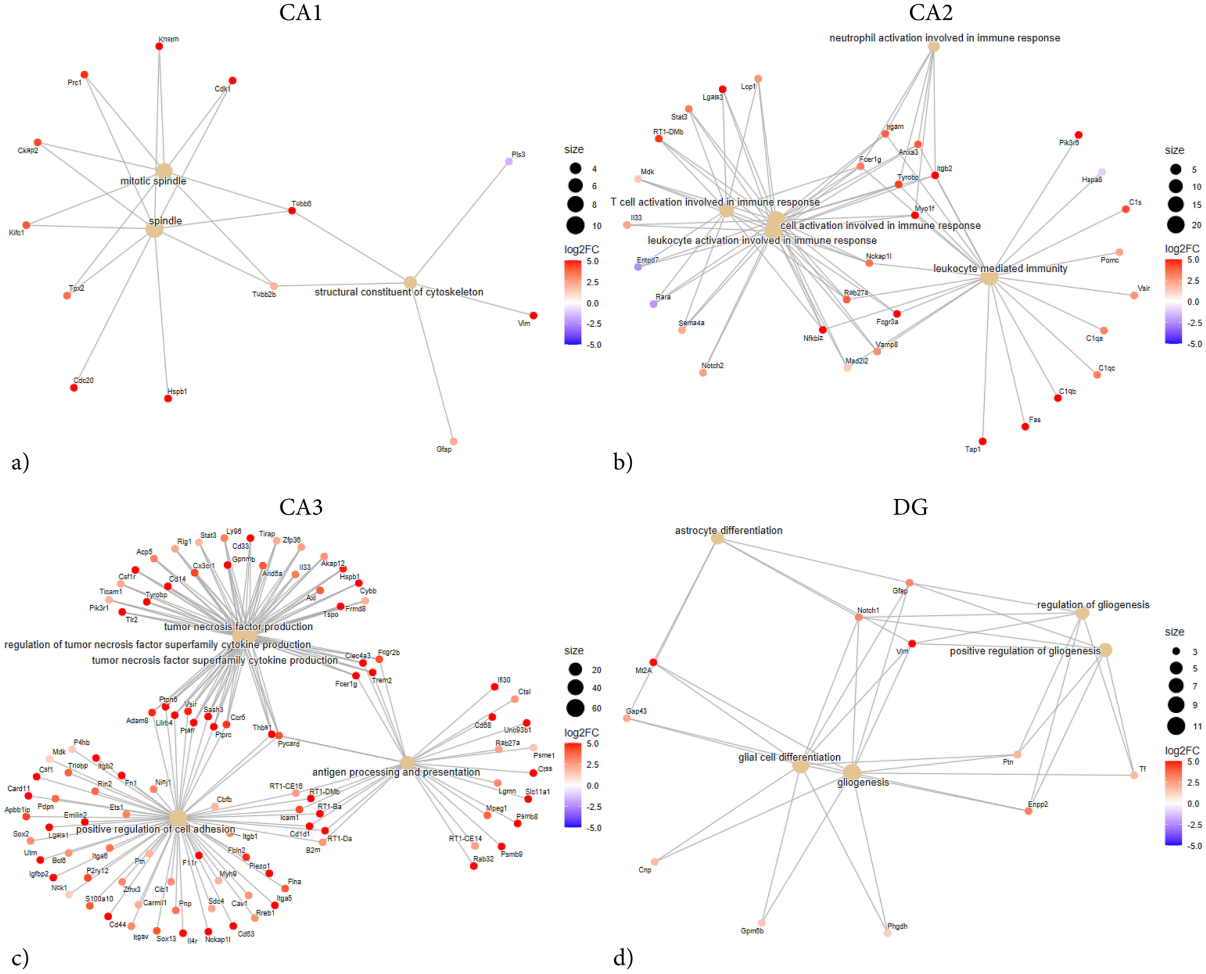
ORA network plots illustrating subregion-specific gene enrichment following penetrating TBI. Over-representation analysis (ORA) network plots showing enriched biological processes and associated genes in hippocampal subregions CA1 **(a)**, CA2 **(b)**, CA3 **(c)**, and DG **(d)** 72 h post-injury. ORA was performed using GO terms. Input genes were limited to those meeting the significance criteria (adjusted *p* < 0.05, log_2_ fold-change > 1), up to 1,000 genes. Enrichment *p*-values were adjusted using Benjamini–Hochberg correction. Node size corresponds to the number of genes associated with each term, and node color reflects log₂ fold change (log_2_FC). The CA3 subregion exhibited the most extensive enrichment, dominated by inflammatory and immune-related terms such as tumor necrosis factor production and positive regulation of cell adhesion. CA2 showed a similar but more confined inflammatory response, including neutrophil activation and leukocyte-mediated immunity. In contrast, CA1 and DG displayed comparatively modest enrichment, with CA1 involving cytoskeletal organization (mitotic spindle, structural constituent of cytoskeleton) and DG showing glial-associated terms such as gliogenesis and astrocyte differentiation. Collectively, these networks illustrate the pronounced and regionally distinct transcriptional activation following pTBI, with the strongest immune signaling localized to CA3 and CA2. CA1 (Sham *n* = 4, TBI *n* = 4), CA2 (Sham *n* = 3, TBI *n* = 4), CA3 (Sham *n* = 3, TBI *n* = 4), and dentate gyrus (DG; Sham *n* = 4, TBI *n* = 3). CA, cornu ammonis; DG, dentate gyrus; FC, fold-change; ORA, over-representation analysis; TBI, traumatic brain injury.

**Figure 6:**
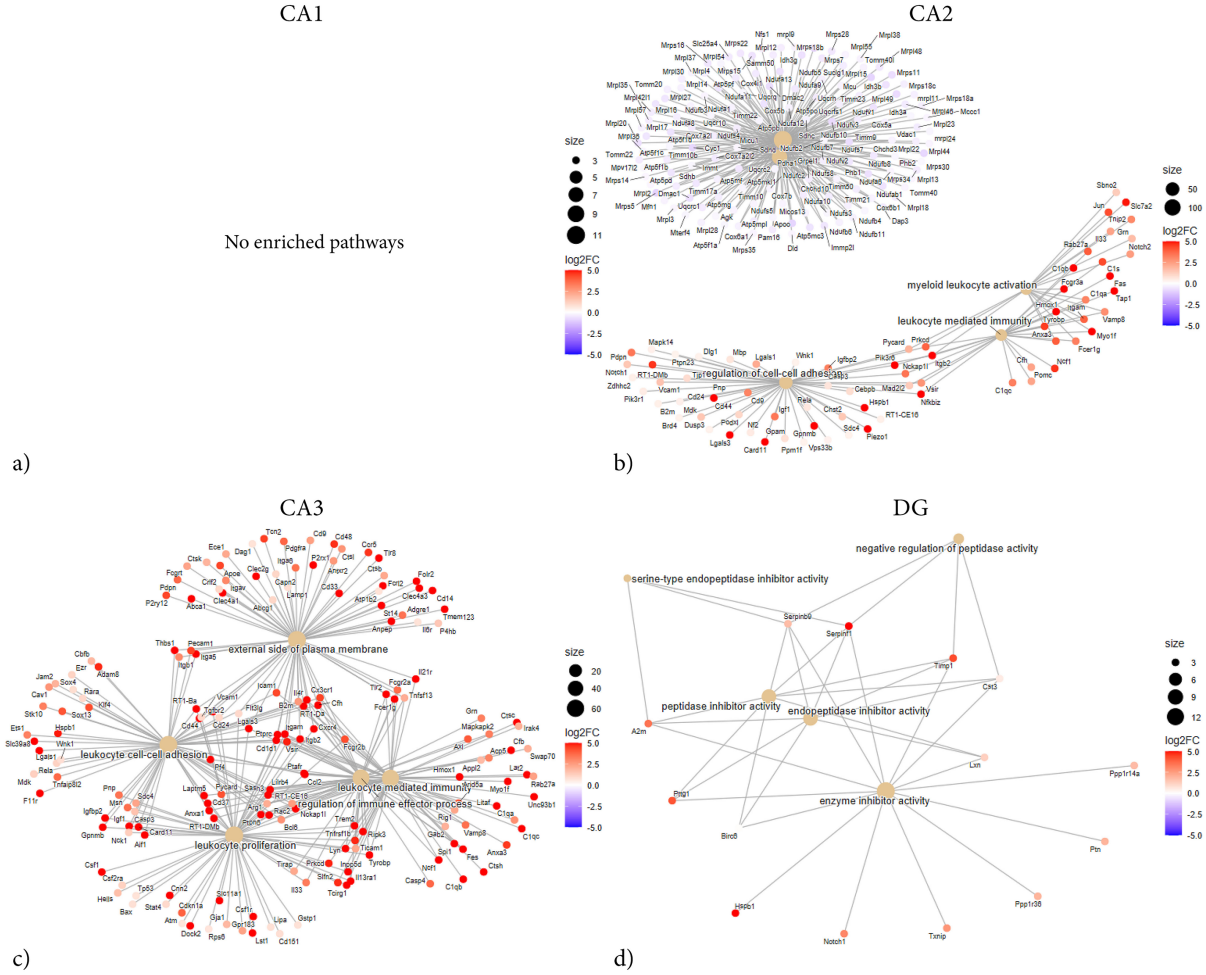
GSEA network plots showing subregion-specific enrichment following penetrating TBI. Gene set enrichment analysis (GSEA) network plots of hippocampal subregions CA1 **(a)**, CA2 **(b)**, CA3 **(c)**, and DG **(d)** 72 h post-injury. GSEA was conducted using fgsea with gene sets derived from the GO knowledgebase. Parameters included a minimum gene set size of 15 and a maximum of 500, with significance set at *p* < 0.005 after Benjamini–Hochberg correction. Node size reflects the number of genes per term, and node color indicates log₂ fold change (log_2_FC). CA1 showed no significant enrichment, indicating a limited transcriptional response. CA2 and CA3 displayed broad enrichment dominated by immune-related processes. In addition, CA2 showed mitochondrial and oxidative stress–related enrichment, suggesting metabolic perturbation alongside inflammation. CA3 exhibited the densest immune network, while DG showed enrichment in peptidase and enzyme inhibitor activity, indicating localized regulatory changes. Overall, GSEA revealed strong inflammatory and metabolic activation in CA2 and CA3, contrasting with the minimal responses in CA1 and DG. CA1 (Sham *n* = 4, TBI *n* = 4), CA2 (Sham *n* = 3, TBI *n* = 4), CA3 (Sham *n* = 3, TBI *n* = 4), and dentate gyrus (DG; Sham *n* = 4, TBI *n* = 3). CA, cornu ammonis; DG, dentate gyrus; GO, gene ontology; GSEA, gene set enrichment analysis; FC, old-change; TBI, traumatic brain injury.

**Figure 7:**
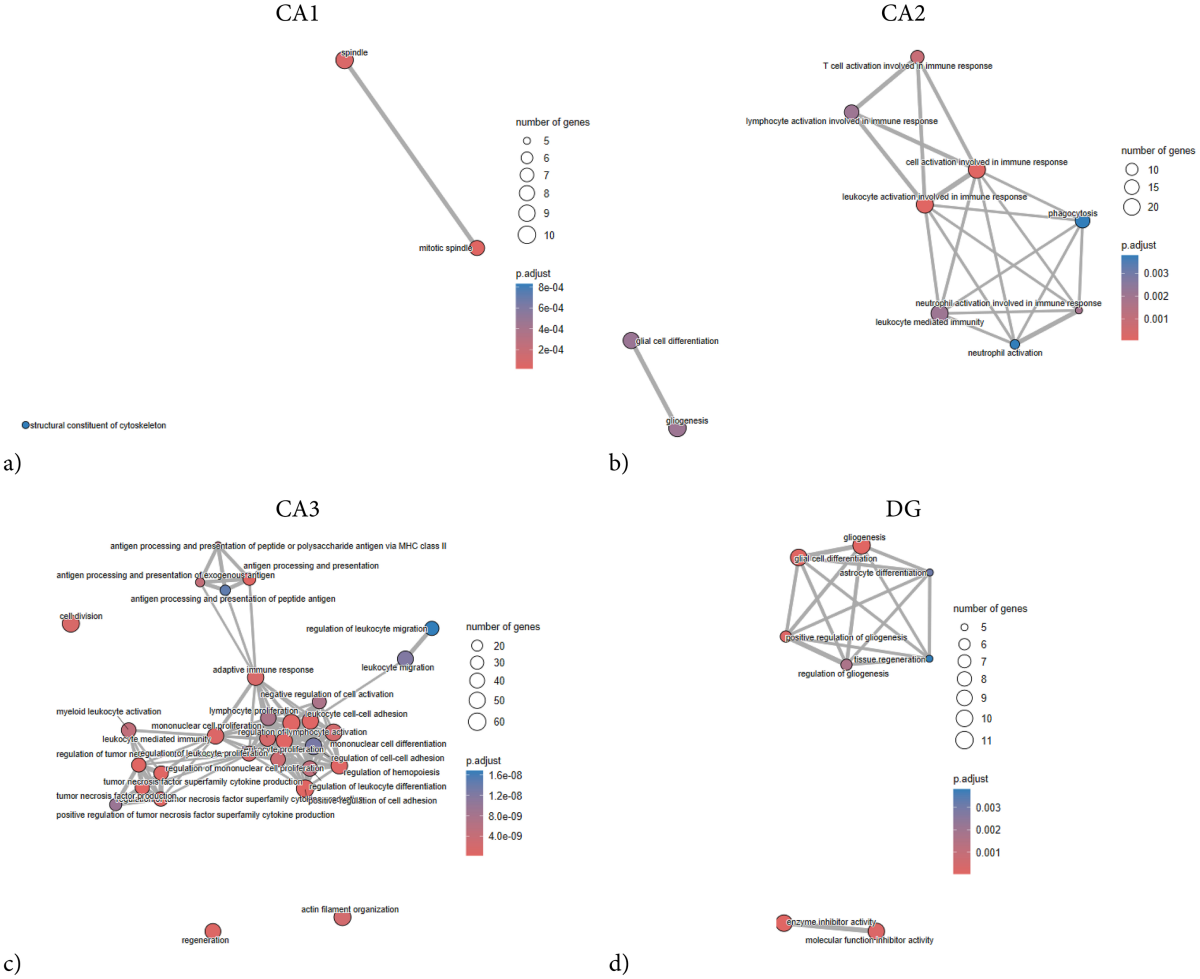
ORA enrichment plots of hippocampal subregions following penetrating TBI. Over-representation Analysis (ORA) network plots showing significantly enriched biological processes in hippocampal subregions CA1 **(a)**, CA2 **(b)**, CA3 **(c)**, and DG **(d)** 72 hours post-injury. ORA was conducted using gene sets derived from the GO knowledgebase. Input genes were limited to those meeting the significance criteria (adjusted *p* < 0.05, log_2_ fold-change > 1), up to 1000 genes. Gene sets containing between 15 and 500 members were tested, and enrichment *p*-values were adjusted via the Benjamini–Hochberg method. Node size corresponds to the number of genes per term, and node color represents the adjusted *p*-value (p.adjust). CA1 displayed limited enrichment restricted to cytoskeletal and mitotic processes, indicating a minor structural or proliferative response. CA2 and CA3 exhibited extensive enrichment dominated by immune-related terms, reflecting pronounced neuroinflammatory activation. The DG showed a smaller but distinct enrichment profile involving gliogenesis, astrocyte differentiation, and enzyme inhibitor activity, consistent with glial and reparative signaling. Overall, ORA revealed strong immune and inflammatory activation in CA2 and CA3, modest glial-associated responses in DG, and minimal transcriptional activity in CA1. CA1 (Sham *n* = 4, TBI *n* = 4), CA2 (Sham *n* = 3, TBI *n* = 4), CA3 (Sham *n* = 3, TBI *n* = 4), and dentate gyrus (DG; Sham *n* = 4, TBI *n* = 3). CA, Cornu Ammonis; DG, Dentate Gyrus; FC, fold-change; ORA, Over-representation analysis; TBI, traumatic brain injury.

**Figure 8:**
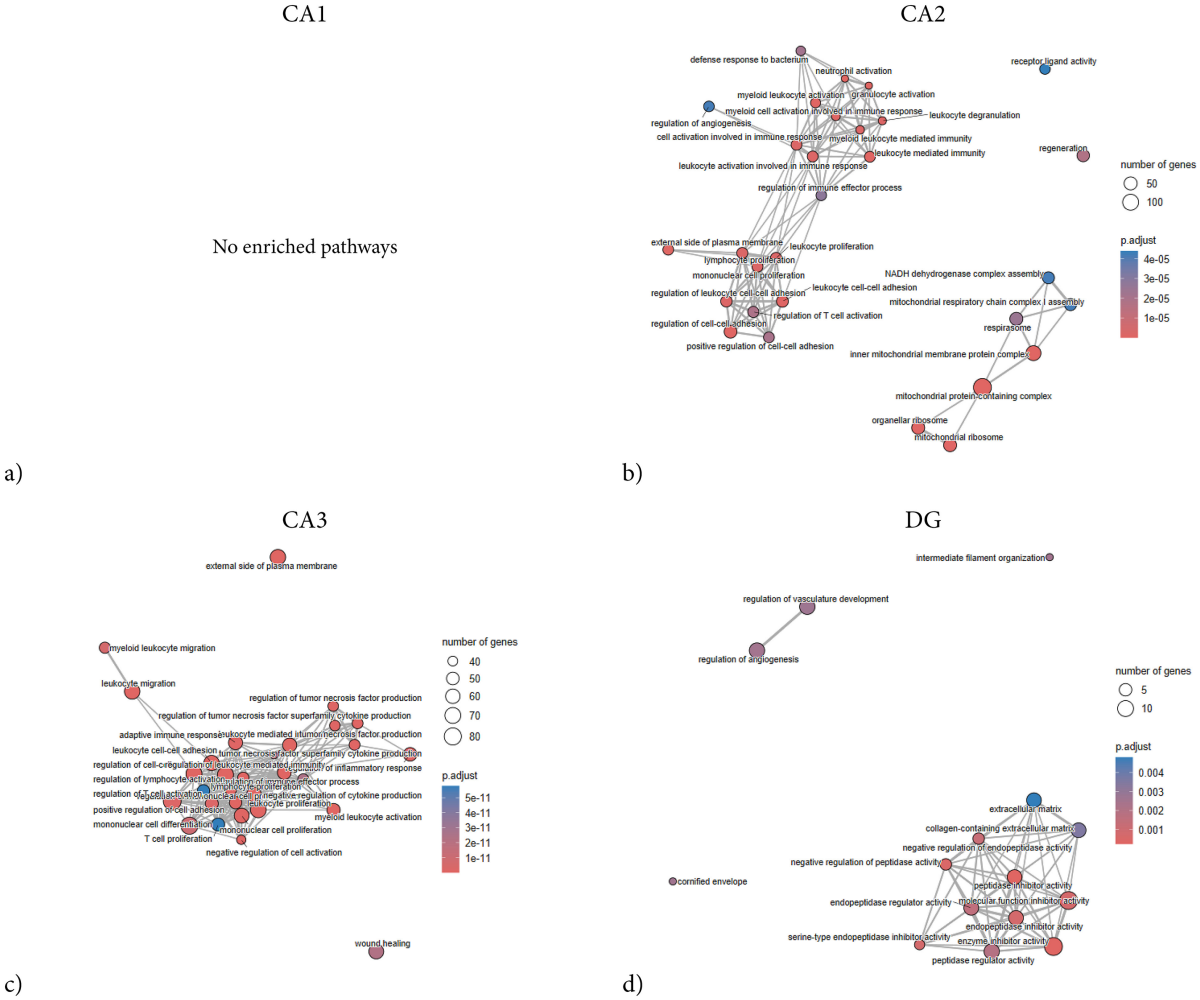
GSEA enrichment plots of hippocampal subregions following penetrating TBI. Gene Set Enrichment Analysis (GSEA) network plots illustrating significantly enriched biological processes and molecular functions in hippocampal subregions CA1 **(a)**, CA2 **(b)**, CA3 **(c)**, and DG **(d)** 72 hours post-injury. GSEA was conducted using fgsea with gene sets derived from the GO knowledgebase. Parameters included a minimum gene set size of 15 and a maximum of 500, with significance set at *p* < 0.005 after Benjamini–Hochberg correction. Node size represents the number of genes per term, while node color indicates adjusted *p*-values (p.adjust). CA1 showed no significant enrichment, indicating minimal coordinated transcriptomic response. In contrast, CA2 and CA3 displayed extensive enrichment dominated by immune- and inflammation-related processes, including leukocyte activation, T cell activation, cytokine production, and regulation of immune effector processes. CA2 further exhibited mitochondrial and oxidative metabolism–related terms, such as mitochondrial respiratory chain complex assembly and NADH dehydrogenase complex assembly, reflecting metabolic stress. The DG showed a distinct enrichment profile centered on peptidase inhibitor activity and angiogenic regulation, suggesting limited but specific molecular adaptations. Overall, CA2 and CA3 demonstrated the most pronounced transcriptional activation, while CA1 remained transcriptionally quiescent at this subacute stage. CA1 (Sham *n* = 4, TBI *n* = 4), CA2 (Sham *n* = 3, TBI *n* = 4), CA3 (Sham *n* = 3, TBI *n* = 4), and dentate gyrus (DG; Sham *n* = 4, TBI *n* = 3). CA, Cornu Ammonis; DG, Dentate Gyrus; GO, Gene Ontology; GSEA, Gene Set Enrichment Analysis; FC, Fold-change; TBI, traumatic brain injury.

**Figure 9:**
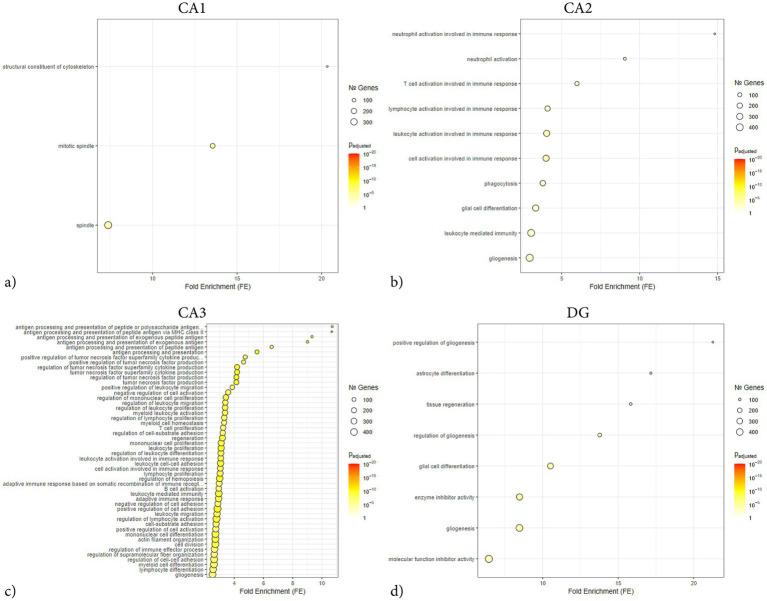
ORA dot plots of enriched GO terms across hippocampal subregions following penetrating TBI. Dot plots show significantly enriched GO knowledgebase terms identified by over-representation analysis (ORA) in laser-captured hippocampal subregions: CA1 **(a)**, CA2 **(b)**, CA3 **(c)**, and DG **(d)** 72 hours post-injury. Input genes were limited to those meeting the significance criteria (adjusted *p* < 0.05, log_2_ fold-change > 1), up to 1000 genes. Gene sets containing between 15 and 500 members were tested, and enrichment *p*-values were adjusted via the Benjamini–Hochberg method. The x-axis represents fold enrichment, dot size indicates the number of genes per GO term, and color scale corresponds to adjusted *p*-value. Marked subregional differences were observed, with CA3 exhibiting a large transcriptomic response with broad enrichment across inflammatory categories, whereas CA1 exhibited few enriched terms, indicating a comparatively muted transcriptional activation. CA1 (Sham *n* = 4, TBI *n* = 4), CA2 (Sham *n* = 3, TBI *n* = 4), CA3 (Sham *n* = 3, TBI *n* = 4), and dentate gyrus (DG; Sham *n* = 4, TBI *n* = 3). CA, Cornu Ammonis; DG, Dentate Gyrus; GO, Gene Ontology; ORA, Over-representation analysis; TBI, traumatic brain injury.

**Figure 10:**
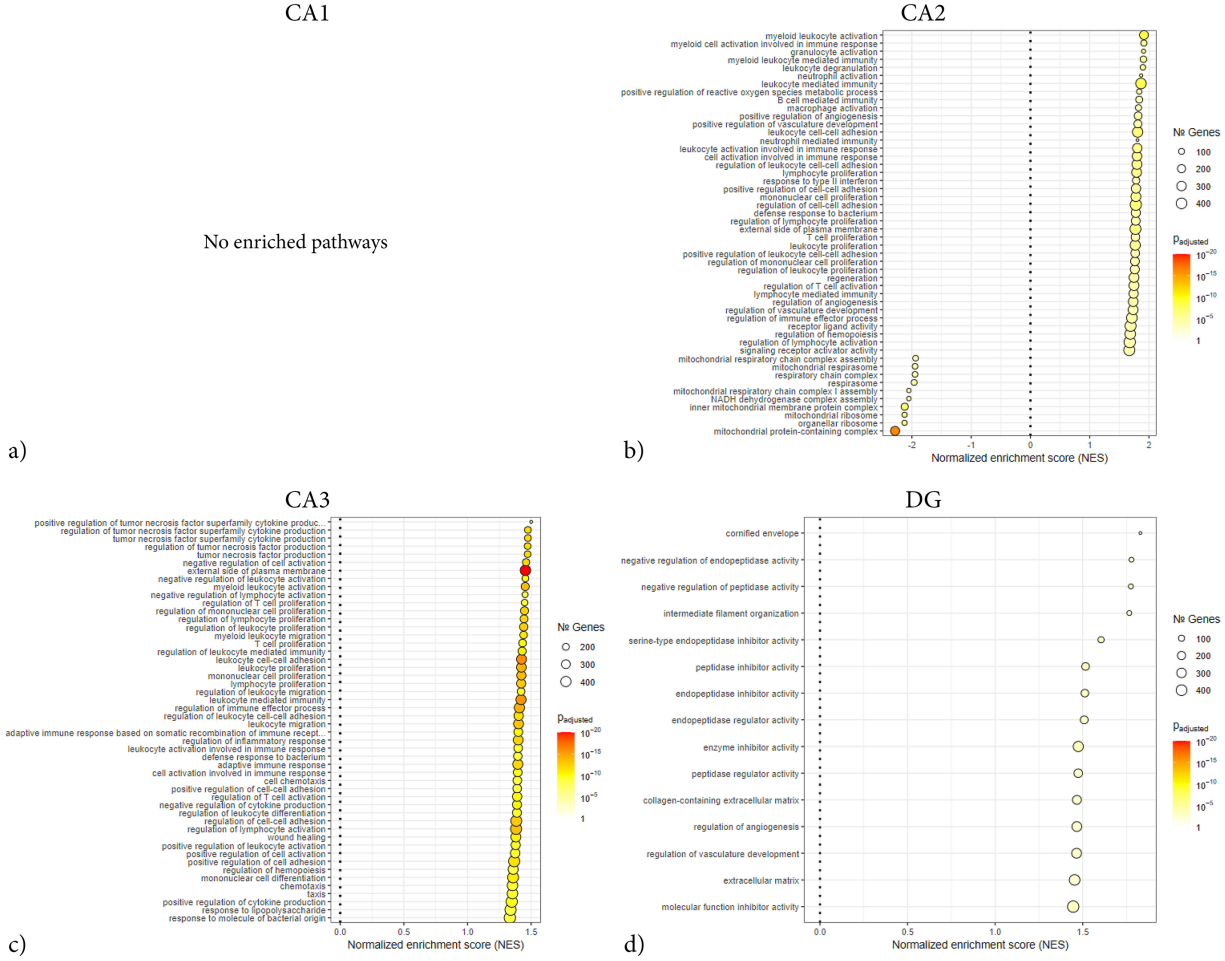
GSEA dot plots of enriched GO terms across hippocampal subregions following penetrating TBI. Dot plots display significantly enriched GO terms identified by GSEA in laser-captured hippocampal subregions: CA1 **(a)**, CA2 **(b)**, CA3 **(c)**, and DG **(d)** 72 hours post-injury. GSEA was conducted using fgsea with gene sets derived from the GO knowledgebase. Parameters included a minimum gene set size of 15 and a maximum of 500, with significance set at *p* < 0.005 after Benjamini–Hochberg correction. The *x*-axis shows the normalized enrichment score (NES), dot size indicates the number of genes contributing to each term, and color represents the adjusted p-value. CA2 and CA3 exhibited extensive enrichment of inflammatory and stress-related pathways, whereas CA1 showed no enrichment whilst DG showed minimal enrichment, consistent with a weaker transcriptional response. Notably, CA2 demonstrated additional enrichment of oxidative phosphorylation and mitochondrial function terms, reflecting subregion-specific stress adaptations. CA1 (Sham *n* = 4, TBI *n* = 4), CA2 (Sham *n* = 3, TBI *n* = 4), CA3 (Sham *n* = 3, TBI *n* = 4), and dentate gyrus (DG; Sham *n* = 4, TBI *n* = 3). CA, Cornu Ammonis; DG, Dentate Gyrus; GO, Gene Ontology; GSEA, Gene Set Enrichment Analysis; NES, Normalized enrichment score; TBI, traumatic brain injury.

Network analysis demonstrated that the DEGs segregated into distinct modules corresponding to functional themes (Figure 5A). One prominent module consisted of tightly co-expressed cell-cycle regulators Cdk1 and Cdc20 —which clustered together and aligned with the mitotic spindle GO category. In parallel, a second module was enriched for cytoskeletal and glial-associated genes, including intermediate filament genes (Vim, Gfap) as well as microtubule components (Tubb2b, Tubb6) (Figure 5A). In contrast, GSEA did not reveal any significant pathway enrichments or coherent gene modules (Figure 6A). The ORA network analysis and enrichment plot were concordant, highlighting the same functional themes (Figure 7A). In line with the GSEA network analysis, the corresponding GSEA enrichment plot showed no significant enrichment of gene sets (Figure 8A). Consistent with the ORA network findings, the ORA dot plot further supported these enrichments (Figure 9A). Finally, the complementary GSEA dot plot likewise showed no significant enrichment (Figure 10A).

#### 3.3.2 CA2

A total of 331 DEGs were identified in CA2, of these 276 were upregulated and 55 were downregulated. Prominent upregulated genes included Itgb2 (integrin subunit β2, LFC = 11.4), Prc1 (protein regulator of cytokinesis 1, LFC = 12.2), Cdk1 (cyclin-dependent kinase 1, LFC = 11.8), Hspb1 (heat shock protein family B member 1, LFC = 8.5), and Nfkbiz (NF-κB inhibitor zeta, LFC = 9). Several structural and extracellular matrix (ECM) associated genes were also strongly induced, including Vim (vimentin, LFC = 6.4), Timp1 (TIMP metallopeptidase inhibitor 1, LFC = 4.3), and Lgals3 (galectin-3, LFC = 9.0). Notable downregulated transcripts included Rara (Retinoic acid receptor alpha, LFC = − 2.2), and Igf1r (IGF-like family receptor 1, LFC = −2.9). The distribution of DEGs is visualized in the CA2 volcano plot (Figure 1b).

GSEA revealed that DEGs in CA2 were significantly enriched in categories associated with immune and inflammatory activation. The strongest positive enrichment scores were observed for inflammatory terms such as “myeloid leukocyte activation,” “granulocyte activation,” “B cell mediated immunity,” “macrophage activation,” and “positive regulation of cell–cell adhesion.” Additional upregulated categories included “positive regulation of angiogenesis,” “T cell activation,” and “positive regulation of reactive oxygen species metabolic process.” In contrast, a set of mitochondrial-related categories showed negative enrichment, including “mitochondrial protein-containing complex,” “NADH dehydrogenase complex assembly,” and “respiratory chain complex assembly,” indicating coordinated downregulation of genes linked to mitochondrial respiration and energy metabolism (Figures 8b, 10b). ORA also exhibited distinct pro-inflammatory regulation, but notably no enrichments regarding mitochondrial function or metabolic regulation (Figures 5b, 7b, 9b).

The GSEA network plot of CA2 DEGs showed clear modular organization (Figure 6b). One major module contained immune and inflammatory genes, including Itgb2, Lgals3, Fcgr3a, Myo1f, Tap1, and Hspb1, strongly connecting to terms such as myeloid leukocyte activation, neutrophil activation, and leukocyte-mediated immunity. Multiple classical-pathway complement components (C1qa, C1qb, C1qc, C1s) were upregulated and formed part of the leading-edge in enriched immune ontologies (myeloid/leukocyte-mediated immunity), consistent with complement engagement. A second major module was formed by mitochondrial ribosomal and respiratory chain genes (e.g., Mrpl, Ndufa, Timm family members), which were predominantly downregulated and associated with terms such as mitochondrial protein-containing complex and respiratory chain complex. A third, smaller network was composed of genes involved in cell adhesion and extracellular signaling, including Vcam1, Cd44, and integrin-associated genes, aligning with enriched categories such as regulation of cell–cell adhesion. Collectively, the network analysis suggests that the CA2 transcriptional response to TBI is dominated by upregulated immune and leukocyte activation modules, alongside a coordinated downregulation of genes related to mitochondrial function.

#### 3.3.3 CA3

CA3 exhibited the largest number and most asymmetrical distribution of DEGs following TBI, a total of 824 of which 811 were upregulated and 13 downregulated (Figure 1c). Among the most strongly upregulated transcripts were Itgb2 (integrin β2, LFC = 14.3), Lgals3 (Galectin-3, LFC = 12), Gpnmb (Glycoprotein nmb, LFC = 14.3), and Trem2 (Triggering receptor, LFC = 7.1), all central mediators of myeloid activation and phagocytosis. Upregulation of C1qa (Complement C1q A chain, LFC = 2.9), C1qb (Complement C1q B chain, LFC = 5.7), and Cfh (Complement factor H, LFC = 6) indicated strong complement system engagement, consistent with innate immune activation after injury. Stress- and injury-related genes such as Hspb1 (heat shock protein family B, LFC = 4.9), Mt1 (Metallothionein 1, LFC = 4.6), Mt2a (Metallothionein 2A, LFC 4.2), and Timp1 (Tissue inhibitor of metalloproteinases-1, LFC = 4.6) were also upregulated, consistent with oxidative stress and ECM remodeling. Downregulated genes in CA3 were associated with impaired ion homeostasis and neuronal excitability. These included Scn1b (sodium channel subunit *β*1, LFC = − 1.8), Trpc6 (transient receptor potential channel, LFC = − 3.7), and Tubb4a (β-tubulin Iva, LFC = − 1.7).

ORA revealed strong enrichment of immune and inflammatory biological processes (Figures 7c, 9c). Top GO terms relate to antigen processing and presentation, cytokine production, leukocyte activation/migration, and cell adhesion. Similarly, several terms involving tumor necrosis factor (TNF) and cytokine production are enriched, including “positive regulation of TNF superfamily cytokine production” and “regulation of inflammatory response.” Terms related to leukocyte migration and activation are prominently enriched, such as “myeloid leukocyte migration” and “lymphocyte activation,” consistent with recruitment and activation of immune cells in CA3. Processes involving cell–cell or cell-matrix adhesion also increase, such as “positive regulation of cell adhesion” and “cell-substrate adhesion,” in line with the upregulation of adhesion molecules. Subsequent GSEA analyses are in line with ORA results, with high normalized enrichment scores for cytokine production and signaling, immune effector process regulation, and cell surface receptor-related processes (Figure 10c). For instance, GSEA identified “regulation of TNF production” and “external side of plasma membrane” as highly enriched gene sets – the latter indicating that many upregulated genes encode cell-surface proteins such as receptors and antigen presentation molecules. GSEA also highlights adaptive immune responses, alongside innate immune activation, suggesting involvement of lymphocytes in addition to myeloid cells. Together, the ORA and GSEA results consistently point to a dominant immune activation signature in CA3, including antigen presentation via MHC-II, pro-inflammatory cytokine signaling, chemotaxis and leukocyte migration, and upregulation of adhesion and membrane proteins that mediate immune cell interactions (Figure 8C).

Network analysis exhibits a similar immune-related theme, consisting of terms related to antigen processing and presentation, all connected by shared upregulated genes such as Cd74, RT1-D, and MHC genes (Figures 5c, 6c). A second cluster centers on cytokine production and immune signaling – terms like TNF production, regulation of cytokine secretion, and inflammatory response group together, indicating that a set of upregulated genes including Tnf, Il1b, and Tyrobp among others contribute to these processes. A third cluster relates to cell adhesion and cell activation; terms such as leukocyte cell–cell adhesion, positive regulation of cell adhesion, and negative regulation of cell activation are linked, driven by upregulated genes encoding adhesion molecules and cell interaction proteins such as Itgb2, Icam1, Vcam1 and Fn1.

Overall, the network analysis highlights that the injury-induced transcriptional program in CA3 is dominated by an immune/inflammatory hub – characterized by antigen presentation and phagocytic activity, heightened cytokine production, and enhanced cell–cell adhesion interactions – all of which facilitate recruitment and communication of immune cells.

#### 3.3.4 DG

A total of 54 DEGs were identified in the DG, with 51 upregulated and 3 downregulated (Figure 1d). Top upregulated transcripts included genes associated with inflammation and injury response. For example, Serpinf1 (Serpin family F member 1, LFC = 9.7), Timp1 (TIMP metallopeptidase inhibitor 1, LFC = 4.28), S100a4 (S100 calcium-binding protein A4, LFC = 6.3), S100a6 (S100 calcium-binding protein A6, LFC = 6.3), Igfbp2 (IGF-binding protein 2, LFC = 6.5), Mt2a (Metallothionein 2A, LFC = 5), Hspb1 (Heat shock protein family B1, LFC = 7.1), and Casp4 (Caspase 4, LFC = 3.7), all exhibited substantial upregulation.

GSEA revealed several enriched genes related to protease inhibition and ECM regulation. In particular, multiple sets associated with enzyme inhibitor activity were significantly enriched after TBI, including “serine-type endopeptidase inhibitor activity,” “peptidase inhibitor activity,” and “enzyme inhibitor activity” (Figures 8d, 10d). Core genes driving these enrichments were various serpins and metalloproteinase inhibitors – for instance, the serine protease inhibitor Serpinf1 and Serpinb9, as well as Timp1 and A2m (alpha-2-macroglobulin) (Figure 6d). Other enriched GSEA terms included those related to ECM structure, such as “collagen-containing matrix,” as well as terms involved in angiogenesis. Notably, no gene sets corresponding to classical inflammatory/immune responses, such as cytokine-mediated signaling or complement activation were enriched.

Enriched GO terms from ORA largely fell into two thematic groups: glial cell activation/development and enzyme inhibition (Figures 7d, 9d). Several ontology terms involving glial proliferation and differentiation were overrepresented, including “positive regulation of gliogenesis,” “astrocyte differentiation,” and “glial cell development.” Key genes contributing to these terms were astrocyte-related and stem cell regulatory genes such as Gfap, Vim, Notch1, and Ptn (Figure 5d). Likewise, ORA confirmed a strong enrichment for “enzyme inhibitor activity,” echoing the GSEA outcome (Figure 7d). Together, the enrichment analyses from both GSEA and ORA indicate that the TBI-induced gene expression program in the dentate gyrus is characterized by reactive glial markers and regulation of tissue remodeling factors.

## 4 Discussion

In this study, we provide a comprehensive transcriptomic analysis of hippocampal subregions following pTBI. Subregional analysis using laser-capture microdissection and subsequent RNA-sequencing reveals pronounced spatial heterogeneity in hippocampal gene expression after pTBI. Collectively, our results reveal subregion-specific differences in transcriptomic activity, gene expression signatures, and pathway enrichments in response to injury. In each subregion, the upregulated DEGs vastly outnumbered downregulated DEGs, highlighting a general pattern of gene induction in the injured hippocampus. Such a predominance of upregulated genes at subacute stages is well established in experimental TBI models and reflects the extensive transcriptional activation associated with secondary injury mechanisms (32, 36). This transcriptional surge coincides with the peak of the neuroinflammatory response, gliosis, and vascular remodeling, representing a coordinated attempt at both damage containment and repair (37–39). The consistency of this pattern across diverse TBI models, including the relatively mild controlled cortical impact and fluid percussion models, suggests that the predominance of upregulated DEGs observed here likely reflects this conserved molecular activation phase following injury.

The penetrating injury model produces a focal cavity with a surrounding penumbra while also generating a pressure wave that propagates throughout the brain. Consequently, all hippocampal subfields are exposed to transient pressure changes and mechanical stress, although regions with strong anatomical connectivity, such as through the fornix, are likely to experience amplified secondary strain due to stress transmission along axonal pathways (33, 34). Consequently, disruption of long-range tracts such as the fornix, or local associative projections, may elicit differential patterns of neuronal stress, activity loss, and compensatory signaling across subregions, thereby shaping the observed transcriptional heterogeneity (40–42). Despite shared exposure to biomechanical perturbation, CA2 and CA3 exhibit distinct molecular responses that likely reflect intrinsic subregional differences rather than differences in injury severity alone. CA3’s dense recurrent excitatory circuitry and high metabolic demand may predispose it to inflammatory and excitotoxic signaling, whereas CA2’s unique synaptic organization and calcium-handling properties may favor stress-adaptive or metabolic suppression responses (20, 43, 44). Taken together, subregion-specific connectivity and intrinsic transcriptional programs likely shape how similar mechanical inputs are translated into divergent molecular phenotypes.

The injury lesion evolves over time, with early edema followed by progressive degeneration that expands over several weeks, ultimately leading to partial loss of the analyzed tissue (34, 45, 46). Transcriptomic analysis was performed 3 days post-injury, corresponding to the subacute phase of secondary injury. This period represents a critical therapeutic window in which inflammatory activation, oxidative stress, and mitochondrial dysfunction are prominent but have not yet progressed to irreversible tissue degeneration (38, 45, 47). Importantly, this time point allows sufficient opportunity for injured tissue and resident cells to mount, adjust, and begin to mature coordinated transcriptional responses, processes that would be incompletely captured at earlier, immediate post-injury time points. Experimental studies demonstrate that interventions targeting inflammation or oxidative stress within the first 72 h reduce lesion expansion and improve functional outcome in rodent models, and we have previously shown that anti-inflammatory treatment attenuates cavity progression in this model (48–51). Thus, the transcriptomic signatures captured at this time point likely reflect active, evolving secondary injury mechanisms that are amenable to modulation, rather than the immediate early gene response or the later degenerative phase in which therapeutic opportunities are substantially diminished.

### 4.1 Method selection and biological variability

The choice of bulk RNA-sequencing over single-cell methods presented both advantages and limitations. RNA-seq provides high sensitivity and broad dynamic range, while laser-capture microdissection preserved precise anatomical resolution and generated neuron-enriched samples, though minimal glial contamination was unavoidable. Hippocampal neuronal layers within each subregion are composed of multiple excitatory principal neuron subtypes with distinct transcriptional and functional profiles, which may influence bulk RNA-seq-derived signatures (52, 53). While LCMD enriches for pyramidal and granule neurons, residual heterogeneity among excitatory subtypes may contribute to averaged expression patterns. Importantly, approximately 95% of the pyramidal layer consists of neurons, a dominance confirmed by our immunofluorescent analyses (Figure 3) (54, 55). The examined regions showed predominantly neuronal staining, with only sparse GFAP-positive astrocytic processes extending into the pyramidal layer, while other glial and vascular markers were confined to adjacent areas. These GFAP-positive elements represent distal astrocytic processes rather than astrocyte somata, as no cell bodies were observed within the captured areas. Fine astrocytic processes contain minimal cytoplasmic volume and low total mRNA abundance (56, 57). Moreover, perisynaptic astrocytic processes are enriched for synaptic and metabolic functions rather than inflammatory gene programs, limiting their potential contribution to immune-related signatures (57). Notably, markers commonly regarded as glial, including GFAP and TSPO, are also expressed at low abundance in neurons, particularly following injury, and inflammatory mediators such as Toll-like receptors and TNF-*α* contribute to neuron-intrinsic inflammatory signaling (58–61). Taken together, these observations indicate that the sequenced material primarily reflects neuronal populations. Nevertheless, sparse astrocytic processes may contribute low-level astrocytic transcripts, and the resulting profiles therefore represent integrated, neuron-enriched subregional responses rather than purely neuronal transcriptional programs, an important consideration when interpreting glia-associated gene expression signatures. While single-cell RNA-seq could theoretically achieve cell-type–specific resolution, the limited amount of dissected tissue and the high neuronal content of these regions made bulk RNA-seq the most robust approach, ensuring deep coverage while preserving regional specificity.

Differential expression analysis applied strict thresholds (log₂ fold change > 1 and adjusted *p* < 0.05). While this increased confidence in the robustness of detected genes, it may underrepresent more subtle yet biologically relevant transcriptional changes. Modest regulation of key signaling or regulatory genes, as well as coordinated but small shifts across gene networks, can nevertheless play important roles in post-traumatic processes such as inflammation, metabolic adaptation, and synaptic remodeling. To address this, we complemented ORA, which relies on predefined cutoffs, with GSEA, which considers ranked expression data independent of thresholding. The combined use of ORA and GSEA therefore allowed us to capture both strongly dysregulated genes and more nuanced, pathway-level trends, providing a more comprehensive view of the transcriptional responses across hippocampal subregions. The concordance between ORA and GSEA supports the robustness of the identified biological themes while capturing both strong gene-level effects and more nuanced pathway-level regulation.

### 4.2 Shared response

Although the hippocampal subregions exhibited fundamentally distinct transcriptomic profiles after penetrating TBI, a small set of stress-associated genes—TSPO, S100a4, Hspb1, Mt2A, Timp1, and Igfbp2—was consistently upregulated across all fields, indicating a conserved core response to injury. These genes represent central elements of the secondary injury cascade, reflecting activation of mitochondrial stress, oxidative imbalance, cytoskeletal instability, and ECM regulation. TSPO, a canonical marker of mitochondrial dysfunction and oxidative stress present in both neurons and glia, was elevated throughout the hippocampus, suggesting a global disturbance of mitochondrial homeostasis (60, 62). Hspb1 and S100a4, both induced in all subregions, mediate cytoskeletal stabilization and chaperone-assisted repair, counteracting protein denaturation and calcium-driven structural collapse (63, 64). The consistent increase of Mt2A reflects an antioxidant defense aimed at sequestering metal ions and limiting oxidative injury, while Timp1 and Igfbp2 indicate coordinated extracellular and trophic adaptations that help preserve matrix integrity and promote cell survival signaling (65–67). Collectively, this gene set defines a unified hippocampal cellular stress module integrating mitochondrial, cytoskeletal, redox, and extracellular protection mechanisms during the early secondary phase of injury. Despite this conserved transcriptional foundation, few enriched pathways were shared between subregions in the overall ontology analyses, underscoring that while the hippocampus mobilizes a common stress-defense machinery at the gene level, the broader biological context diverges sharply. CA1, CA2, CA3, and DG each extend this core program along distinct trajectories, ranging from structural stress signaling to inflammatory, metabolic, or reparative activation. These networks converge on core components of the secondary injury cascade, including inflammation, oxidative stress, and gliosis, which together shape the distinct molecular landscape of each hippocampal subregion and are examined in detail in the following sections.

### 4.3 Neuroinflammatory response

**Figure 11:**
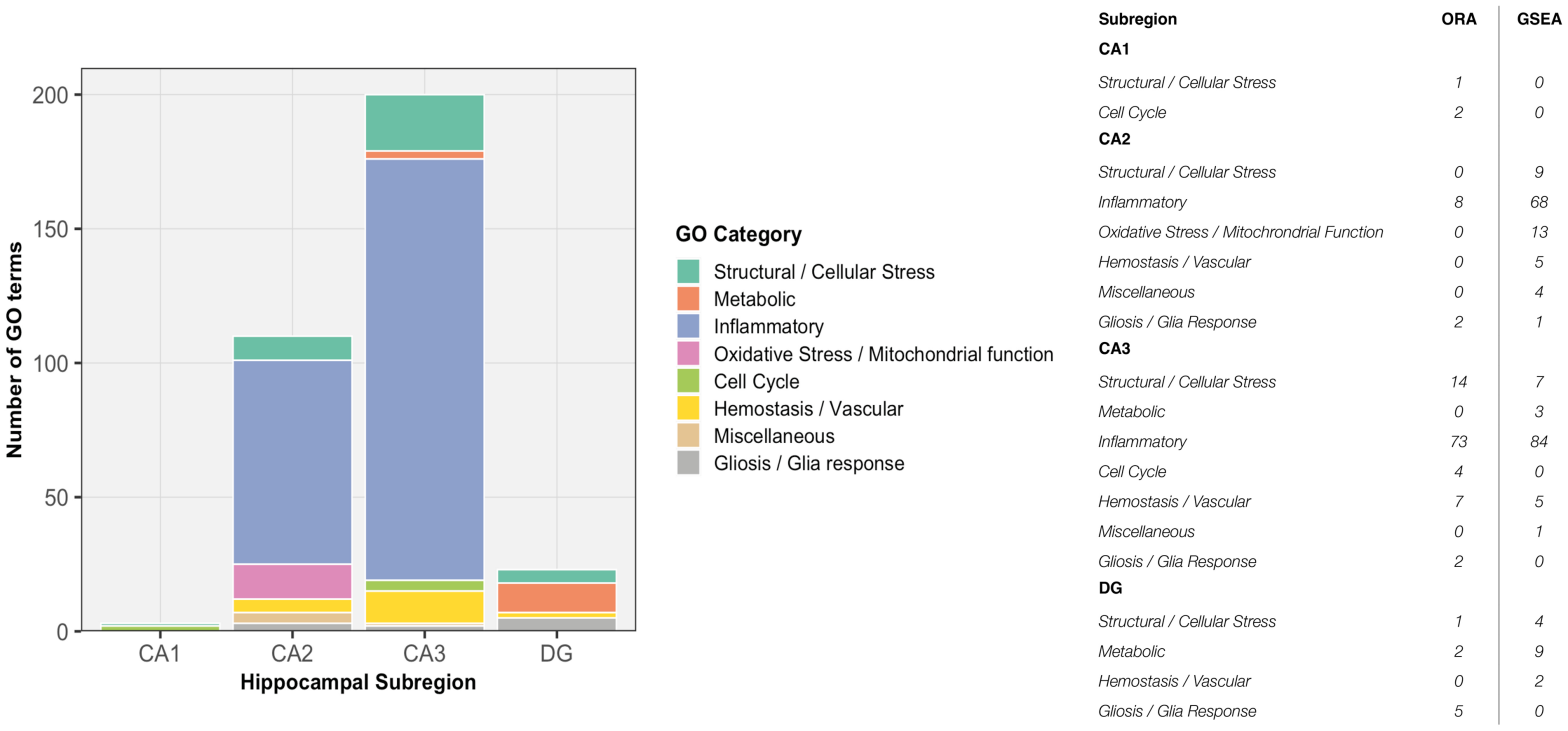
Subregion-specific enrichment of GO terms following penetrating TBI. Stacked bar plots illustrate the number and categorical distribution of significantly enriched GO terms across hippocampal subregions (CA1, CA2, CA3, DG) derived from over-representation analysis (ORA) and gene set enrichment analysis (GSEA). GO terms were manually grouped into eight functional categories reflecting dominant biological processes. CA2 and CA3 displayed markedly greater enrichment compared to CA1 and DG, with CA3 characterized by a strong inflammatory signature and CA2 showing both inflammatory and oxidative stress/mitochondrial dysfunction profiles, indicating distinct stress-response mechanisms among hippocampal subfields. CA1 (Sham *n* = 4, TBI *n* = 4), CA2 (Sham *n* = 3, TBI *n* = 4), CA3 (Sham *n* = 3, TBI *n* = 4), and dentate gyrus (DG; Sham *n* = 4, TBI *n* = 3). CA, cornu ammonis; DG, dentate gyrus; GO, gene ontology; GSEA, gene set enrichment analysis; ORA, over-representation analysis; TBI, traumatic brain injury.

A key finding of the present study was the region-specific inflammatory response across hippocampal subfields following pTBI. This aligns with the well-established subacute neuroinflammatory peak typically observed within the first week of injury (8). However, our results reveal distinct subregional patterns: CA2 and CA3 displayed strong and diverse inflammatory signatures, whereas CA1 and DG were devoid of enriched inflammatory pathways (Figure 11). The lack of inflammatory activation in CA1 and DG contrasts with previous reports describing widespread microglial and astrocytic responses in these subfields within 1–3 days post-injury (68, 69). The pyramidal and granule cell layers, which were selectively isolated in this study, are densely populated by neurons and contain relatively few glial or vascular cells (54, 55). As a result, our neuron-enriched design largely excluded transcripts from microglia, astrocytes, and endothelial cells, the primary mediators of cytokine signaling, leukocyte adhesion, and complement activation. Accordingly, the absence of inflammatory enrichment in CA1 and DG likely reflects the limited inflammatory response in CA1 and DG neurons rather than a true absence of immune activity within the tissue. Collectively, these findings indicate that the inflammatory milieu reported in previous studies originates mainly from non-neuronal compartments.

Although both CA2 and CA3 exhibited robust transcriptional activation of immune pathways, their inflammatory programs diverged markedly, indicating subfield-specific mechanisms of neuronal immune engagement. Increasing evidence suggests that injured neurons can express complement components, cytokine modulators, and adhesion molecules, enabling them to participate actively in early innate immune responses following trauma or ischemia, consistent with our findings (70–73). In CA2, the transcriptional response was dominated by genes involved in innate immune activation, complement signaling, and cell-adhesion processes. Enriched ontologies such as myeloid leukocyte activation, leukocyte–cell adhesion, and complement cascade point to a primarily innate, chemotactic inflammatory state within the pyramidal neuronal population. This transcriptional pattern is consistent with early complement engagement and adhesion-mediated recruitment of immune effectors, mechanisms known to facilitate microglial contact, synaptic surveillance, and localized remodeling rather than widespread inflammatory damage (74–79). The upregulation of complement components together with integrin signaling molecules and Fc-receptor family genes, suggests that CA2 neurons participate in the early immune–synaptic interactions. Such activity-dependent complement tagging has been linked to selective pruning of weakened synapses and synaptic homeostasis during both development and injury and may thus represent an attempt to stabilize circuitry after TBI. This interpretation aligns with emerging evidence that CA2 neurons possess intrinsic resilience to injury compared to neighboring CA1 and CA3 regions (43).

In contrast, the CA3 subfield exhibited a broader and more complex inflammatory signature, encompassing cytokine signaling, TNF-superfamily activation, and MHC class II antigen-presentation pathways, indicating a shift toward a mixed innate–adaptive neuronal immune response. This pattern is consistent with previous observations of robust hippocampal inflammation and microglial activation in the subacute phase after TBI, although most prior studies have focused on glial rather than neuronal populations (80–82). The present findings extend this literature by suggesting that CA3 neurons transcriptionally engage in immune signaling, implying that neurons are not merely passive targets of glial cytokines but actively shape the inflammatory environment through expression of immune mediators and antigen-processing machinery. Although blood–brain barrier (BBB) disruption occurs in our injury model, and regional differences in BBB permeability have been reported, the pronounced neuronal immune activation observed in CA3 likely reflects intrinsic subregional properties rather than a primarily vascular-driven effect (34, 83). CA3 pyramidal neurons form dense recurrent excitatory networks and receive powerful glutamatergic input from the DG, conferring a high degree of synaptic plasticity and metabolic demand. These same features also render CA3 highly susceptible to excitotoxic and cytokine-induced stress, where heightened excitatory drive amplifies inflammatory cascades (84–86). Functionally, such neuronal engagement in cytokine and TNF signaling could have implications for synaptic stability and hippocampal output. TNF-*α* and IL-1β are known to modulate glutamatergic transmission and dendritic spine morphology, shifting the excitatory–inhibitory balance and altering network synchronization (87–89). In the context of CA3’s recurrent circuitry, these processes could lead to hyperexcitability, impaired pattern completion, and reduced circuit resilience, ultimately contributing to cognitive and memory dysfunction observed after TBI. Thus, while CA2 appears to engage in complement-mediated synaptic refinement, CA3 demonstrates a pro-inflammatory and cytokine-driven activation pattern that may disrupt normal circuit communication and amplify secondary injury progression.

### 4.4 Mitochondrial dysfunction and oxidative stress

Our findings in the CA2 subregion revealed a strong signature of mitochondrial dysfunction, detected by GSEA but not ORA, suggesting that this response results from coordinated, moderate transcriptional shifts across multiple mitochondrial pathways rather than isolated, high-amplitude gene changes (90). The enriched GSEA terms included oxidative phosphorylation, mitochondrial respiratory chain complex assembly, ATP metabolic process, mitochondrial translation, and NADH dehydrogenase complex assembly, all showing negative enrichment scores. This indicates a broad suppression of mitochondrial bioenergetic and structural functions, encompassing both reduced oxidative metabolism and downregulation of genes related to mitochondrial organization and biogenesis. These features suggest that CA2 neurons enter a state of metabolic and structural restraint rather than activating repair or biogenic pathways.

This widespread downregulation aligns with established models of secondary injury after TBI, in which excitotoxic calcium influx and ischemia disrupt electron transport at mitochondrial complexes I and III, resulting in reduced ATP synthesis, membrane depolarization, and increased ROS formation (91–93). The coordinated suppression of oxidative-phosphorylation and NADH-linked pathways in CA2 likely reflects an adaptive reduction in metabolic flux, aimed at minimizing oxidative injury and stabilizing intracellular calcium homeostasis during the acute phase.

This transcriptional signature was unique to CA2, highlighting the region’s distinctive metabolic and physiological profile. CA2 neurons are enriched for mitochondrial and calcium-handling machinery, including the mitochondrial calcium uniporter complex, and exhibit dense dendritic mitochondrial networks that tightly couple synaptic activity to oxidative metabolism (43, 94, 95). This architecture sustains high energetic throughput, but following injury it may predispose CA2 mitochondria to calcium-driven overload and depolarization. The observed downregulation of mitochondrial and respiratory-chain pathways therefore likely represents a controlled metabolic rebalancing, a temporary suspension of oxidative phosphorylation to limit ROS generation and preserve organelle integrity. Together with its high mitochondrial capacity and robust calcium-buffering mechanisms, these features likely provide CA2 with a larger metabolic reserve, allowing neurons to withstand energy downscaling without immediate degeneration (43, 94).

While CA2 is often considered one of the most resilient hippocampal subfields, direct evidence of its oxidative vulnerability remains limited. Under physiological conditions, its enhanced antioxidant and calcium-buffering potential likely confer resistance to excitotoxic and ischemic injury (43). However, these same features may render CA2 highly responsive to mitochondrial stress, as its dense mitochondrial population and tight calcium–metabolic coupling are energetically demanding (95). The observed suppression of mitochondrial structural and functional pathways therefore likely represents a regulated protective mechanism, aimed at conserving energy and preventing oxidative damage rather than indicating irreversible dysfunction. Nevertheless, prolonged energetic depression would inevitably constrain ATP production, impair ion homeostasis, and reduce synaptic efficacy (96). Given CA2’s crucial role in social memory and temporal coding, such bioenergetic limitations could attenuate its excitatory output to CA1 and disrupt hippocampal network synchrony, leading to subtle but persistent cognitive deficits even without significant cell loss.

### 4.5 Gliosis

The hippocampal subregions displayed distinct transcriptional profiles reflecting differences in reparative capacity after injury. The DG showed selective activation of processes related to gliosis, tissue regeneration, and vascular remodeling, whereas CA1 exhibited a comparatively muted, cell-autonomous response dominated by cytoskeletal organization and cell-cycle regulation. No comparable activation of reparative pathways was detected in CA2 or CA3. As the laser-captured material was enriched for neurons, these results primarily represent neuron-derived transcriptional programs, suggesting that the apparent gliogenesis-associated and angiogenic activity in the DG reflects neuronal engagement of repair-associated signaling networks rather than widespread glial transcription.

In the DG, both GSEA and ORA analyses revealed enrichment of gliogenesis, astrocyte differentiation, ECM organization, regulation of angiogenesis, and enzyme inhibitor activity, together defining a reparative transcriptional state characterized by matrix stabilization, vascular support, and proteolytic control. The enrichment of protease-inhibitory and ECM-related pathways implies that DG neurons contribute to limiting secondary tissue breakdown, maintaining BBB-stability, and creating an environment conducive to microvascular remodeling and neurogenesis (97, 98). Functionally, these processes suggest that the DG enters a protective restructuring phase, aimed at stabilizing the injured microenvironment and facilitating subsequent neuronal and vascular recovery. Although the DG showed gliogenic enrichment, the magnitude of this response was modest, consistent with a low-to-moderate activation pattern rather than a large-scale gliotic shift. This restrained transcriptional activity aligns with recent single-cell and spatial transcriptomic studies, which similarly report limited astroglial proliferation in the DG after injury, alongside prominent neurogenic and niche-stabilizing responses (31, 99, 100). TBI biases neural stem cell fate toward neurogenesis and away from astrogliogenesis, highlighting that early DG remodeling primarily reflects neurogenic and vascular niche activation rather than astrocyte expansion (31). The moderate gliogenesis terms observed in this study are therefore best interpreted as neuron-driven signaling that interfaces with glial and vascular pathways, consistent with a reparative but not proliferative response. Importantly, many genes annotated to gliogenesis, angiogenesis, and ECM-pathways are also induced in neurons following injury, where they contribute to neuron–glia-vascular signaling and tissue repair rather than astrocyte proliferation per se (101, 102). The presence of moderate GFAP enhancement in the DG warrants cautious interpretation. While GFAP splice variants in hippocampal neurons have been described, neuronal GFAP expression remains debated (58, 59, 103, 104). Consequently, the observed GFAP signal may partly be explained by minor astrocytic contamination of the laser-captured tissue rather than genuine neuronal transcription. Nonetheless, such limited contamination does not account for the full range of enriched processes—particularly ECM organization, angiogenesis regulation, and protease inhibition—which indicate a coordinated, neuron-driven reparative response that partially overlaps with glial signaling domains. This composite signature likely represents the early molecular phase of neuron–glia–vascular coordination, consistent with the DG’s established role as the hippocampal hub of post-injury remodeling.

### 4.6 Limitations

Our study has several limitations. The modest sample size per hippocampal subregion reflects the experimental design, which required LCMD of multiple regions from each animal, thereby constraining the number of biological replicates and reducing power to detect transcripts with small effect sizes. In addition, only male animals were included to avoid further reducing the sample size per group, which limits inference regarding sex-specific transcriptional responses. The low RNA input inherent to LCMD-based sampling may also reduce sensitivity for low-abundance transcripts. These limitations were addressed by applying conservative statistical criteria and by emphasizing consistent, biologically coherent transcriptional patterns across subregions.

### 4.7 Summary

In summary, the present transcriptomic analysis reveals striking subregional specialization in the hippocampal response to penetrating TBI. CA2 and CA3 displayed robust yet divergent neuronal inflammatory programs consistent with their distinct circuit properties, with CA2 engaging an innate, complement-associated response suggestive of synaptic stabilization within a metabolically constrained and structurally stable network, whereas CA3 exhibited a cytokine-driven activation profile aligned with its densely recurrent excitatory circuitry and known susceptibility to activity-dependent amplification and excitotoxic stress (43, 44, 105). In parallel, CA2 showed coordinated downregulation of oxidative phosphorylation and mitochondrial biogenesis pathways, consistent with protective metabolic suppression aimed at limiting oxidative injury and preserving organelle integrity. By contrast, the DG mounted a modest but distinct reparative transcriptional program characterized by neuronal engagement of gliogenic, angiogenic, and ECM-pathways, in line with its established role in plasticity, neurogenesis, and circuit remodeling after injury (102). Together, these findings delineate a heterogeneous hippocampal response in which CA2 prioritizes metabolic restraint and synaptic stabilization, CA3 engages pro-inflammatory and stress-amplifying signaling, and the DG initiates neuron-driven repair, collectively highlighting region-specific strategies that shape post-traumatic recovery and vulnerability across the hippocampal circuit. Functionally, these region-specific responses suggest differential circuit consequences, with CA3 inflammation contributing to network instability and impaired pattern completion, CA2 metabolic suppression favoring neuronal survival at the cost of reduced excitatory throughput and DG-driven repair and stabilization of circuitry. From a translational perspective, this subfield-specific framework provides a foundation for developing targeted neuroprotective and immunomodulatory interventions to mitigate neuronal dysfunction and cognitive decline. Future studies including both sexes, integrating single-cell and spatial transcriptomics with validation of key pathways such as mitochondrial suppression in CA2 and extensive inflammatory activation in CA3 will be crucial to link these molecular programs to functional outcomes and to guide precision strategies for limiting secondary injury and promoting circuit restoration after TBI.

## Data Availability

The RNA-sequencing data generated in this study are publicly available in the NCBI Sequence Read Archive (SRA) under BioProject accession PRJNA1402624. Additional data is available upon request.
