# Supplementary material for: Subregional differences in the hippocampal transcriptomic response after penetrating traumatic brain injury in rats
Source: Front Neurol. 2026 Feb 23;16:1729794. doi: 10.3389/fneur.2025.1729794 (PMC12967967; doi:10.3389/fneur.2025.1729794)
Supplement: Supplementary file 1 [file Data_Sheet_1.docx]

Supplementary Material


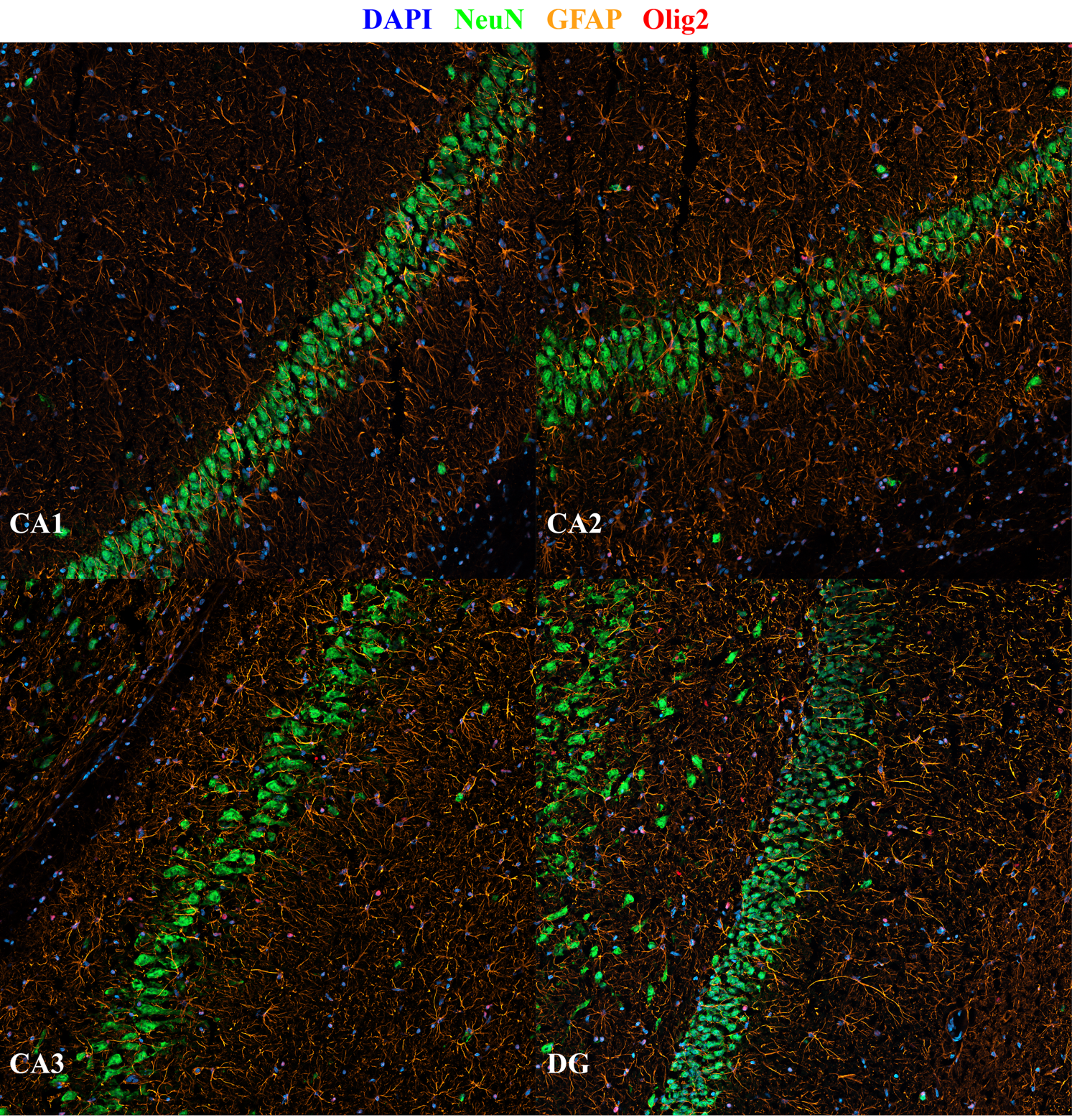


**Supplementary Figure 1. Excerpts from Figure 3 showing neuronal and glial organization across hippocampal subregions.** Immunofluorescent images of hippocampal subregions (CA1, CA2, CA3, and dentate gyrus; DG) stained for DAPI (nuclei, blue), NeuN (neurons, green), GFAP (astrocytes, orange), and Olig2 (oligodendrocytes, red). These excerpts from Figure 3 are shown separately for clarity and emphasize the neuronal composition of the pyramidal and granule cell layers, with GFAP-positive astrocytic processes partly infiltrating but mainly surrounding the neuronal zones. Olig2-positive cells were sparse and localized outside the principal layers, confirming that the laser-captured regions were predominantly neuronal with limited astrocytic contamination and negligible oligodendroglial contribution.


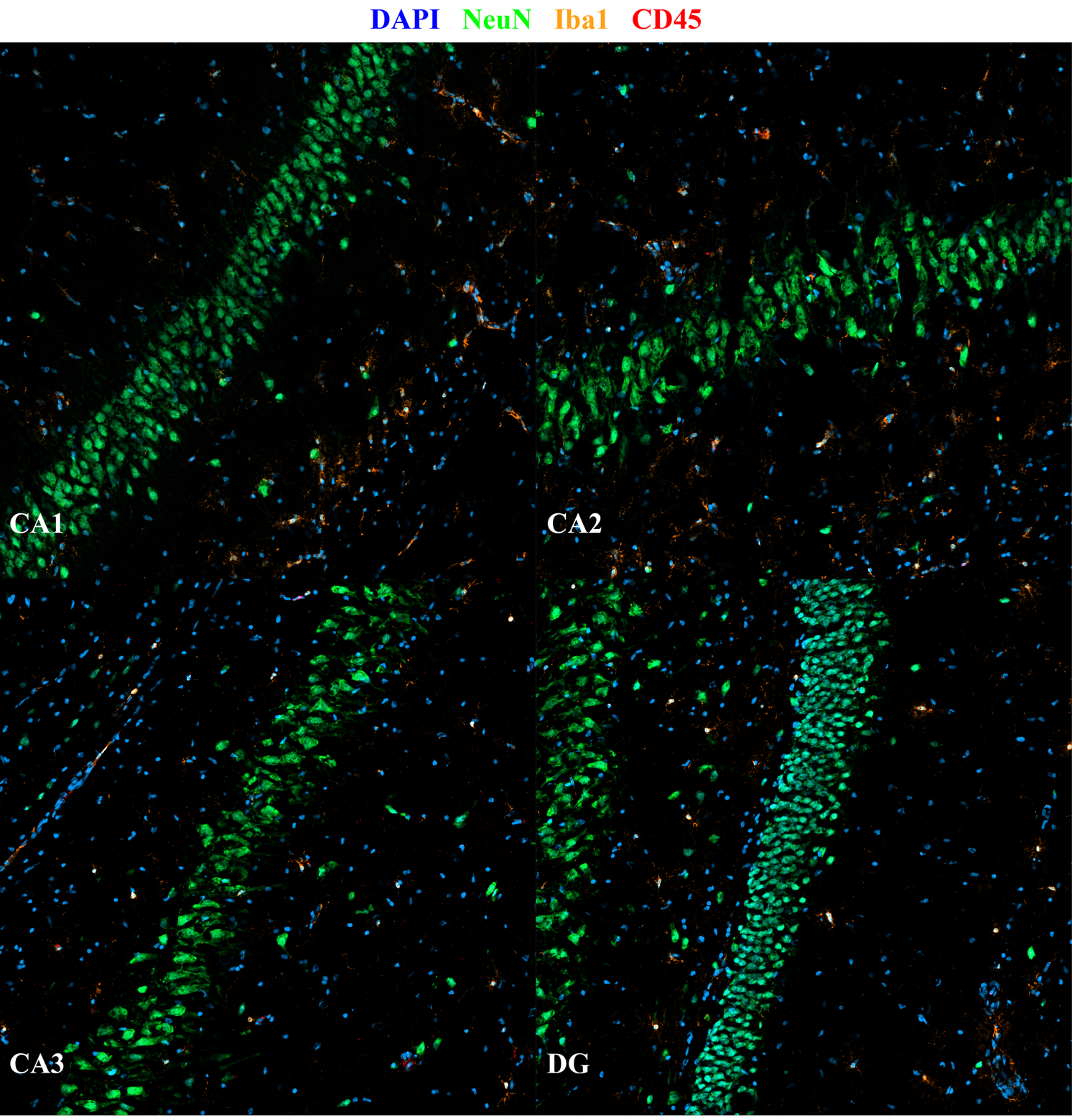


**Supplementary Figure 2. Representative excerpts from Figure 3 showing microglial and immune marker distribution across hippocampal subregions.** Immunofluorescent images of hippocampal subregions (CA1, CA2, CA3, and dentate gyrus; DG) stained for DAPI (nuclei, blue), NeuN (neurons, green), Iba1 (microglia, orange), and CD45 (infiltrating immune cells, red). These excerpts from Figure 3 are presented separately for clarity and illustrate the absence of Iba1- or CD45-positive cells within the pyramidal and granule cell layers, indicating that the laser-captured regions were largely devoid of microglial or infiltrating immune contamination. NeuN labeling delineates the densely neuronal composition of the isolated layers, confirming their specificity for neuronal populations.


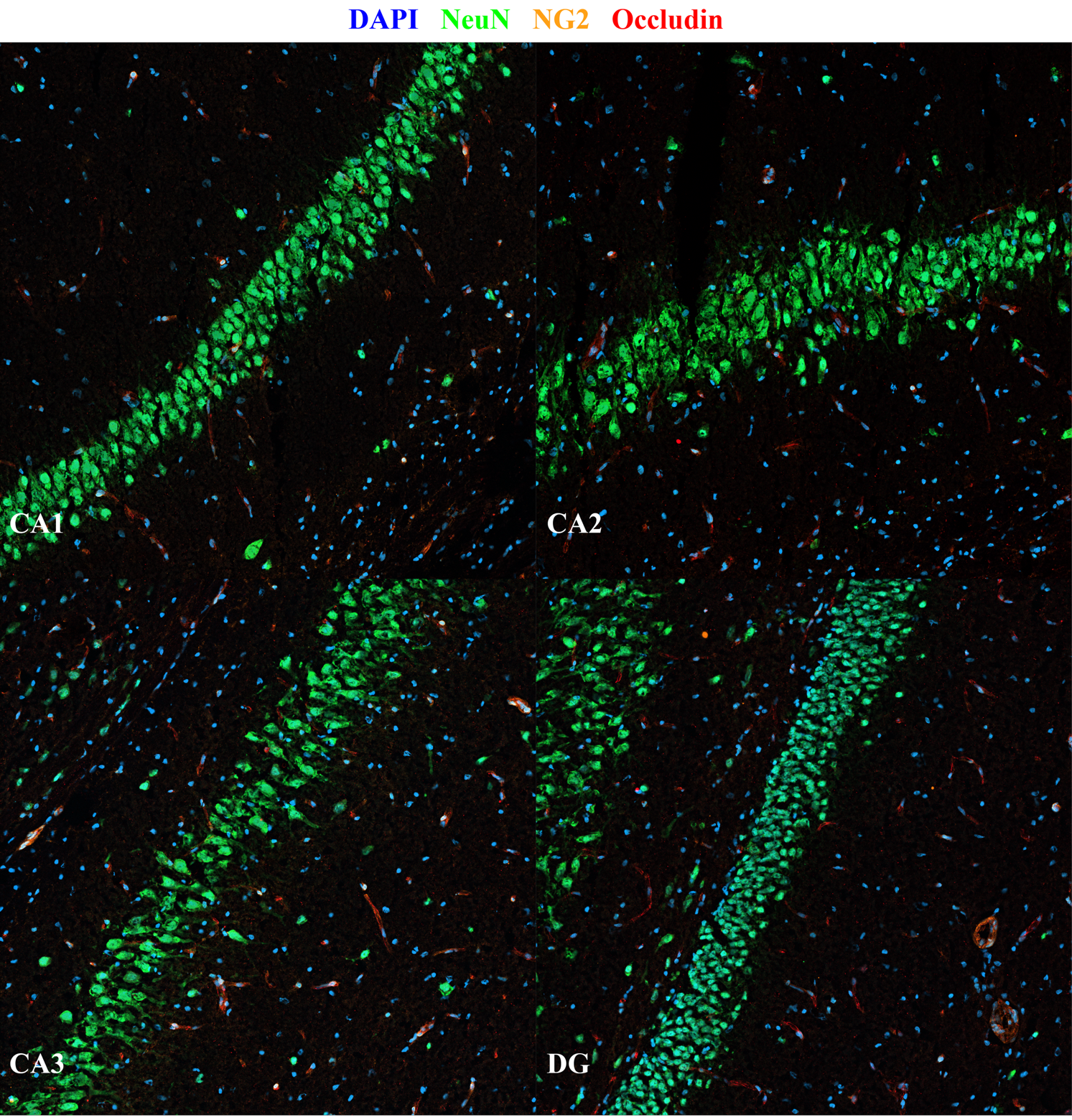


**Supplementary Figure 3. Representative excerpts from Figure 3 showing distribution of vascular and progenitor markers across hippocampal subregions.** Immunofluorescent images of hippocampal subregions (CA1, CA2, CA3, and dentate gyrus; DG) stained for DAPI (nuclei, blue), NeuN (neurons, green), NG2 (oligodendrocyte precursor cells, orange), and Occludin (endothelial tight junctions, red). These excerpts from Figure 3 are displayed separately for clarity and reveal that NG2-positive cells were present only in association with Occludin-positive vascular structures, with no NG2-positive/Occludin-negative cells detected within the pyramidal or granule cell layers. This indicates that the laser-captured regions were free of oligodendrocyte precursor contamination and primarily composed of neuronal populations.
